# Fluorinated Deep Eutectic Gel Electrolytes with Simultaneously Enhanced Mechanical Strength and Ionic Conductivity for Solid‐State Lithium Metal Batteries

**DOI:** 10.1002/adma.74180

**Published:** 2026-07-18

**Authors:** Hao Long, Yuhao Liang, Ting He, Xueming Chen, Zimo Huang, Hao Chen, Shanqing Zhang

**Affiliations:** ^1^ Institute For Sustainable Transformation School of Chemical Engineering and Light Industry Guangdong University of Technology Guangzhou China; ^2^ Guangdong Provincial Laboratory of Chemistry and Fine Chemical Engineering Jieyang Center Jieyang China; ^3^ School of Metallurgy and Environment Central South University Changsha China

**Keywords:** deep eutectic solvent, elastomeric solid‐state electrolyte, gel electrolyte, lithium‐metal battery, molecular competition, phase separation

## Abstract

Gel polymer electrolytes for lithium‐metal batteries face an inherent trade‐off between mechanical strength and ionic conductivity. Herein, we present a molecular‐level strategy that harnesses competitive hydrogen‐bonding interactions to spontaneously generate a nanoscale phase‐separated architecture in the deep eutectic gel (DEG) electrolyte. Through one‐step in‐situ copolymerization of acrylamide and *N,N*‐dimethylacrylamide within a trifluoromethyl‐functionalized deep eutectic solvent comprising *N*‐methyl‐2,2,2‐trifluoroacetamide (TNMA) and lithium bis(trifluoromethanesulfonyl)imide (LiTFSI), an interpenetrating network is formed, in which rigid polyacrylamide‐rich domains reinforce the matrix while polydimethylacrylamide‐rich channels facilitate ion transport. Driven by the competition between polymer–polymer and polymer–solvent hydrogen bonds, the resulting DEG electrolyte achieves an exceptional ionic conductivity of 2.99 mS cm^−1^ at 30°C, an excellent Li^+^ transference number of 0.78, and a remarkable tensile strength of 11.4 MPa with 473% elongation. Meanwhile, TNMA, together with TFSI^−^, regulates the Li^+^ solvation structure and interfacial chemistry, promoting the formation of a LiF‐rich interphase through fluorinated‐solvent‐ and anion‐involved interfacial reactions. The resulting Li||Li symmetric cells operate for over 3500 hours (0.1 mA cm^−2^), and Li|DEG|NCM811 cells retain 77.5% capacity after 400 cycles at 2 C. This work establishes competitive molecular interactions as a design principle for next‐generation gel polymer electrolytes.

## Graphical abstract

The synergistic integration of rigid polyacrylamide (PAM) and flexible poly(*N,N*‐dimethylacrylamide) (PDMA) phases offers exceptional toughness to suppress lithium dendrites, simultaneously enhancing mechanical strength and ionic conductivity.

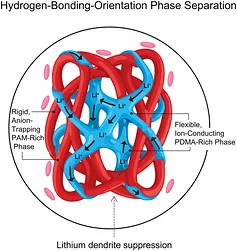

## 1 Introduction

Gel polymer electrolytes (GPEs) represent a promising intermediate between liquid and solid‐state electrolytes, aiming to enable safe, high‐performance lithium‐metal batteries (LMBs) [1, 2, 3, 4, 5]. By immobilizing liquid electrolyte within a polymer matrix, GPEs can theoretically combine the high ionic conductivity of liquids (typically 10^−3^–10^−2^ S cm^−1^) with the mechanical stability and safety of solids. However, conventional GPEs are plagued by a fundamental trade‐off. Increasing the polymer content enhances mechanical strength for dendrite suppression but inevitably reduces ionic conductivity by restricting ion mobility [6, 7]. Conversely, achieving high ionic conductivity necessitates a high content of liquid electrolyte, which often involves volatile and flammable organic solvents that compromise the mechanical integrity and introduce severe safety risks related to leakage and thermal runaway [8, 9]. This inverse relationship has proven largely insurmountable within homogeneous gel structures, demanding a conceptual departure toward systems where mechanical and transport functions are spatially decoupled.

Deep eutectic solvents (DESs) have emerged as attractive, safer electrolyte media, yet translating their excellent ionic transport into mechanically robust deep eutectic gels (DEGs) has been surprisingly elusive. Formed through hydrogen‐bonding interactions between organic donors and acceptors, DESs exhibit negligible vapor pressure, inherent nonflammability, and wide electrochemical windows, all while maintaining ionic conductivities comparable to conventional organic electrolytes [10, 11, 12]. The challenge, however, lies in the very interactions that define them. The extensive hydrogen bond network that stabilizes a DES also solvates polymer chains so effectively that it prevents them from aggregating to form a robust, reinforcing network. Consequently, in situ polymerization within a DES often yields soft, jelly‐like DEGs with insufficient structural integrity to suppress dendrite growth or function as standalone separator layers [13, 14, 15].

Recent advances in tough hydrogels and ionogels have demonstrated that phase‐separated polymer networks can achieve exceptional mechanical properties through synergistic stress dissipation mechanisms. By engineering distinct microphases with complementary functions (one providing elasticity and the other dissipating energy), these materials overcome the traditional stiffness‐toughness trade‐off [16, 17, 18]. However, applying this powerful design principle to DEGs presents unique thermodynamic constraints. The hydrogen‐bonding network inherent to DESs acts as a potent compatibilizer, reducing the interfacial tension between different polymer components and suppressing the entropic driving force for phase separation [19]. The few successful attempts to structure DES gels have relied on complex, multi‐step methods that offer only modest gains [20, 21, 22, 23, 24]. Achieving controlled phase separation while maintaining continuous, high‐mobility ion‐conducting pathways requires a level of molecular engineering that has not yet been realized in DEG systems.

Another significant challenge hindering the application of DEGs in LMBs is the issue of interfacial side reactions. The active hydrogen atoms present in many hydrogen bond donors (e.g., amides, ureas) can undergo parasitic reactions with the highly reductive lithium metal anode [25, 26, 27]. This reaction not only degrades the polymer structure but also leads to the continuous consumption of lithium and the deterioration of the solid electrolyte interphase (SEI), often accompanied by the release of flammable hydrogen gas (H_2_). While strategies like incorporating fluoroethylene carbonate (FEC) can shield these active hydrogens, they often introduce new compromises, such as gas generation at elevated temperatures and depressed mechanical properties [28, 29]. A more integrated molecular design is therefore required to simultaneously instill mechanical robustness and engineer a stable electrode‐electrolyte interface from the ground up. Recently, Zhang et al. reported fluorinated amide‐based deep eutectic gel electrolytes, in which fluorination site and fluorination degree were used as molecular design variables, and amide LUMO level together with the Li^+^ desolvation barrier were proposed as screening criteria for Li‐metal interfacial stabilization [30]. This strategy effectively regulates Li^+^ solvation/desolvation behavior and promotes the formation of an inorganic‐rich interphase. However, integrating the interfacial advantages of fluorinated amides with a controllable phase‐separated polymer network remains an unresolved challenge.

Herein, we present a one‐step strategy that leverages competitive hydrogen bonding to resolve both the mechanical and interfacial challenges in a single, integrated design. By copolymerizing acrylamide (AM), a hydrogen bond donor and acceptor, and *N,N*‐dimethylacrylamide (DMA), an acceptor‐only monomer, within a rationally designed fluorinated DES, we program the system for spontaneous nanoscale phase separation. The solvent component, *N*‐methyl‐2,2,2‐trifluoroacetamide (TNMA), creates a precisely tuned thermodynamic environment where the strong self‐association of AM units can effectively compete with their solvation by TNMA. This molecular competition drives the formation of a bicontinuous architecture that systematically decouples key electrolyte properties. Rigid, polyacrylamide‐rich domains provide exceptional mechanical reinforcement (Young's modulus: 5.14 MPa), while interconnected, solvent‐swollen polydimethylacrylamide‐rich channels maintain high ionic conductivity (2.99 mS cm^−1^). Meanwhile, TNMA, together with TFSI^−^, helps regulate the Li^+^ solvation structure and interfacial chemistry, promoting the formation of a LiF‐rich interphase through fluorinated‐solvent‐ and anion‐involved interfacial reactions. This engineered interphase effectively passivates the lithium metal, preventing parasitic reactions with the electrolyte's hydrogen bond donors. The resulting electrolyte achieves an unprecedented synergy of properties, including a tensile strength of 11.4 MPa, extensive stretchability (473%), a high Li^+^ transference number of 0.78, and stable cycling for over 3500 hours in Li||Li symmetric cells. This work establishes competitive hydrogen bonding as a powerful strategy for creating multifunctional materials, offering a holistic solution to the multifaceted challenges of safety, mechanical integrity, and interfacial stability in next‐generation electrolytes.

## 2 Results and Discussion

### 2.1 Design and Synthesis of Phase‐Separated Deep Eutectic Gels

To overcome the intrinsic coupling between stiffness and conductivity, we developed a molecular competition‐driven strategy to create a bicontinuous, phase‐separated deep eutectic gel (DEG) electrolyte. In our design, two monomers with contrasting hydrogen‐bonding capabilities were copolymerized in situ via UV‐initiated radical polymerization directly within a fluorinated deep eutectic solvent (DES) matrix. Specifically, acrylamide (AM, a strong hydrogen‐bond donor and acceptor) and *N,N*‐dimethylacrylamide (DMA, hydrogen‐bond acceptor only, no donor) were polymerized in a mixture of *N*‐methyl‐2,2,2‐trifluoroacetamide (TNMA) and lithium bis(trifluoromethanesulfonyl)imide (LiTFSI) salt (Figure S1). The TNMA–LiTFSI mixture forms a deep eutectic solvent through Lewis acid–base interactions, where Li^+^ (Lewis acid) coordinates with the carbonyl oxygen (Lewis base) of TNMA. This molecular interaction induces charge delocalization, which significantly depresses the melting point, maintaining the DES in a liquid state at room temperature when the TNMA/LiTFSI molar ratio ranges from 5:1 to 9:1 (Figure S2). Raman spectroscopy further demonstrates progressive salt dissociation with increasing TNMA content, as evidenced by an increase in the fraction of free TFSI^−^ from 62% (5:1 ratio) to 78% (9:1 ratio), with a corresponding decrease in the ion‐pair peaks (Figure S3). Fourier‐transform infrared (FTIR) spectroscopy confirms the TNMA–LiTFSI interactions via characteristic blue shifts in the C═O stretch (from 1565 to 1573 cm^−1^) and N─H stretch (from 3336 to 3360 cm^−1^) upon salt addition, indicating coordination of Li^+^ with the carbonyl and hydrogen bond formation between TNMA and TFSI^−^ (Figure S4). Systematic optimization reveals that a TNMA:LiTFSI molar ratio of 8:1 yields maximum ionic conductivity (4.36 mS cm^−1^ at 30°C), balancing salt dissociation with charge carrier concentration. This optimized composition serves as our standard DES throughout this study (Figure S5). We then introduced AM and DMA monomers into the optimized DES and carried out photopolymerization to form the solid DEG electrolyte (Figure S6). Successful polymerization is verified by Raman and FTIR spectroscopy (Figures S7 and S8), which showed the disappearance of the monomer C═C bonds and shifts in C═O vibrations, confirming that a crosslinked polymer network had formed. As demonstrated in Figures S9 and S10, through process optimization, the deep eutectic gel electrolyte can be prepared to a thickness of 40 µm while ensuring good interfacial contact with the cathode.

**FIGURE 1:**
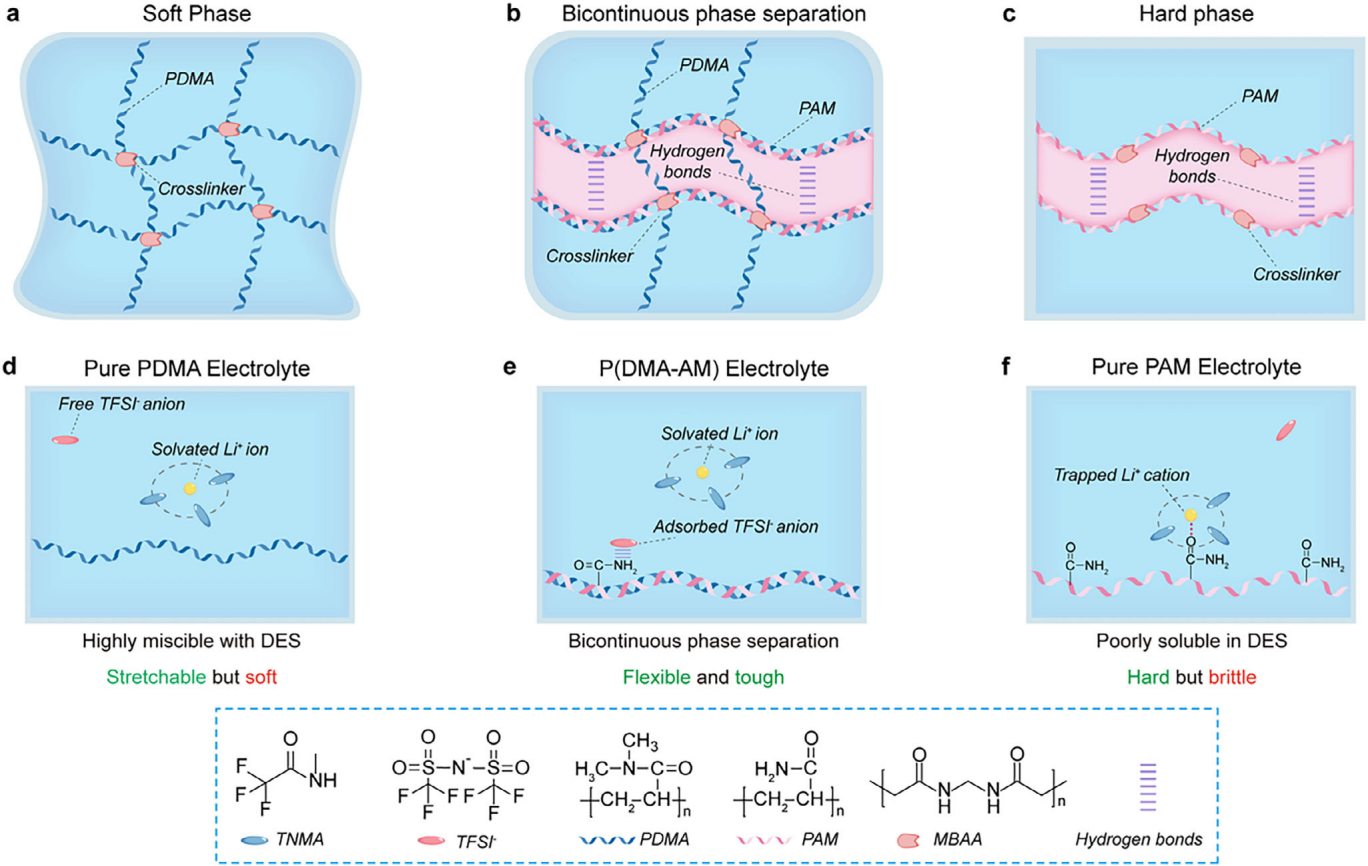
**Schematics of phase‐separated deep eutectic gel (DEG) electrolytes with various AM content**. (a–c) Structural evolution of the polymer networks with varying acrylamide (AM) content: (a) homogeneous soft phase (pure PDMA gel); (b) bicontinuous phase‐separated network (P(AM‐DMA) gel) induced by hydrogen‐bonding competition; and (c) aggregated hard phase domains (pure PAM gel). (d–f) Corresponding ion transport mechanisms: (d) negligible anion‐polymer interaction in the PDMA electrolyte; (e) anchoring anion via hydrogen bonding in the P(AM‐DMA) electrolyte; and (f) Li^+^ trapping caused by the dense carbonyl domains in the pure PAM network. The chemical structures of the solvent components (TNMA, LiTFSI), polymer segments (PDMA, PAM), and crosslinker *N,N'*‐Methylenebisacrylamide (MBAA) are listed at the bottom.

The key innovation of this system lies in exploiting the thermodynamic incompatibility between the two polymer components within the DES. Poly(dimethylacrylamide) (PDMA, from DMA monomers), lacking any hydrogen bond donor functionality, is highly solvated by and miscible with the DES (Figure 1a). In stark contrast, polyacrylamide (PAM, from AM monomers) contains self‐associating amide (─CONH_2_) groups that preferentially form strong intermolecular hydrogen bonds (Figure 1c). During the random copolymerization of AM and DMA in the DES (i.e., P(AM‐co‐DMA)), this disparity in interaction leads to a spontaneous segregation: PAM‐rich segments tend to hydrogen bond with each other and phase‐separate out of the DES, while PDMA‐rich segments remain dissolved in the DES (Figure 1b). The result is analogous to a spinodal decomposition process, yielding an interpenetrating network of two continuous phases comprising rigid PAM‐rich domains and soft PDMA‐rich domains [31]. Unlike conventional single‐phase gels, this bicontinuous architecture inherently decouples mechanical reinforcement from ion‐transport channels. This microstructural plays a key role in regulating the local ionic environment (Figure 1d–f). Unlike the weak interaction in pure PDMA or the Li^+^ trapping seen in dense PAM, the copolymer interface enables an anion‐anchoring effect. Amide groups bind TFSI^−^ anions through hydrogen bonding, which releases Li^+^ and facilitates selective conduction.

**FIGURE 2:**
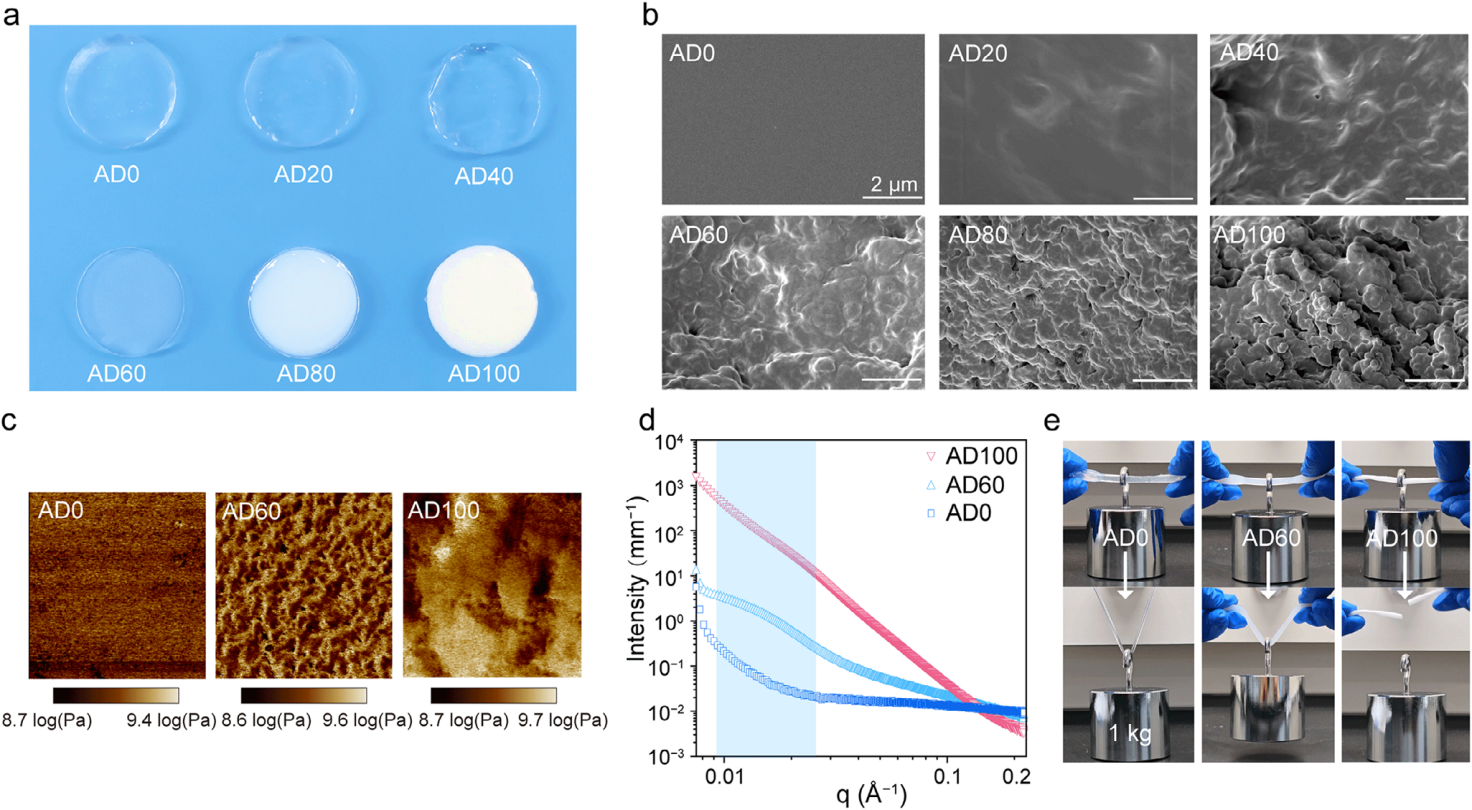
**Characterization of the phase separation and mechanical properties of DEGs**. (a) Photos of DEGs showing a transition from transparent (AD0, i.e., 0% PAM, 100% PDMA) to opaque (AD100, i.e., 100% PAM, 0% PDMA) with increasing AM percentage (shown as in the following number), indicating progressive phase separation. (b) SEM images revealing the microstructural evolution from a smooth, featureless morphology in AD0 to a porous, heterogeneous morphology in AD100. (c) AFM quantitative nanomechanical modulus maps of AD0, AD60, and AD100. The AD60 map confirms a bicontinuous morphology with interwoven soft PDMA‐rich (dark) and stiff PAM‐rich (bright) domains. (d) SAXS profiles of DEGs with different AM fractions, showing a characteristic peak for the phase‐separated AD60. (e) Photos of a load‐bearing test illustrating that the bicontinuous AD60 DEG (cross‐section: 4 mm^2^) can sustain a 1 kg load, whereas the AD0 and AD100 gels fail.

To establish the relationship between composition and phase‐separated structure, we synthesized a series of copolymer DEGs with systematically varied AM:DMA ratios, denoted as AD*x* (where *x* is the molar percentage of AM in the monomer feed). Striking visual and morphological changes were observed as x increased. The gel with no AM (AD0, a pure PDMA network) was optically transparent, indicative of a homogeneous structure lacking obvious light‐scattering domains. As the AM content increased, the gels transitioned from transparent to translucent and eventually opaque, signaling the onset of microphase separation (Figure 2a). Scanning electron microscopy (SEM) of cryo‐fractured surfaces revealed increasingly pronounced fluctuations correlated to the AM content, reflecting the growing structural heterogeneity (Figure 2b). AD0 displays a smooth, featureless surface, consistent with a uniform polymer–solvent matrix. At 40% AM content (AD40), polymer‐rich domains become clearly visible due to the decreased compatibility of the PAM‐rich chains with DES. With the AM content further increasing to 80%, the AD80 displays distinct granular structures and a milky‐white appearance, indicative of submicron‐scale phase transitions (Figure S11). At the highest acrylamide content (AD100, a pure PAM network), the gel shows extensive phase separation and porosity, and the material is brittle due to poor integration with the solvent phase. These observations confirm that increasing the fraction of PAM drives greater phase segregation in the DEG. A detailed investigation into the thermal stability of the deep eutectic gels is provided in Figures S12 and S13. Compared with the nonfluorinated deep eutectic gels, the fluorinated eutectic gels exhibit markedly improved flame retardancy, which is mainly attributed to the presence of the electron‐withdrawing ─CF_3_ group in TNMA that lowers the intrinsic flammability of the amide framework and makes ignition and flame propagation less favorable [32].

We further characterized the emerging microstructure using atomic force microscopy (AFM). Young's modulus maps (Figure 2c) distinguished regions of differing stiffness within the gel surface. AD0 showed a uniformly soft mechanical response, consistent with a homogeneous gel network. In contrast, AD60 exhibited a nanoscale heterogeneous pattern composed of interwoven soft and stiff regions, indicating the coexistence of mechanically distinct domains. AD100 showed predominantly stiff regions with reduced soft‐phase continuity, consistent with large‐scale heterogeneity and decreased DES‐rich continuity. To further correlate this nanoscale heterogeneity with local chemical distribution, atomic force microscopy in combination with infrared‐spectroscopy (AFM‐IR) measurements were performed (Figure S14). The AFM phase image of AD60 showed clear nanoscale phase contrast across the scanned region, indicating spatial variation in the local mechanical/viscoelastic environment. More importantly, the corresponding AFM‐IR chemical map collected at 1610 cm^−^^1^, a PAM‐related amide band, also exhibited a nonuniform spatial distribution of signal intensity over the same region. Localized spectra collected from two representative positions showed distinct intensities of the 1610 cm^−^^1^ band, further supporting local variation in PAM‐related chemical composition. These combined phase and chemical contrasts support nanoscale phase separation involving PAM‐associated heterogeneous domains in AD60.

Small‐angle X‐ray scattering (SAXS) was further used to provide characteristic length‐scale information for this heterogeneous structure (Figure 2d). AD0 showed weak and featureless scattering, consistent with a homogeneous network without distinct nanoscale domains. In contrast, AD60 displayed a broad scattering feature at q = 0.009–0.026 Å^−^^1^, corresponding to characteristic domain spacings of 24.2–69.8 nm based on d = 2π/q. The broadness of this feature indicates a distribution of domain spacings rather than a highly ordered periodic structure. The subsequent rapid in situ polymerization kinetically traps this nonequilibrium network structure, preserving the bicontinuous‐like nanoscale morphology [33]. At higher AM contents, the SAXS intensity increased, but the peak became ill‐defined, suggesting that the polymer‐rich domains tended to coalesce into larger and more irregular structures. Such oversized domains likely compromise the balance of properties by creating isolated polymer‐rich regions and stress concentrators while reducing continuous ion‐transport pathways.

The practical implications of this phase‐separated architecture are dramatically illustrated through mechanical testing (Figure 2e and Video S2). We performed a simple weight‐bearing demonstration by stretching dog‐bone‐shaped specimens (4 mm^2^ cross‐section) of various gels and attaching a 1 kg mass. The pure PDMA DEG (AD0), while highly stretchable, could not support the weight due to insufficient strength. The pure PAM DEG (AD100) was strong but brittle; it snapped without appreciable elongation. Impressively, the bicontinuous copolymer DEG at intermediate composition (AD60) was able to sustain the 1 kg load while remaining intact and elastic, demonstrating the synergistic mechanical enhancement achieved through optimal phase separation.

### 2.2 Hydrogen‐Bonding‐Orientation Phase Separation

**FIGURE 3:**
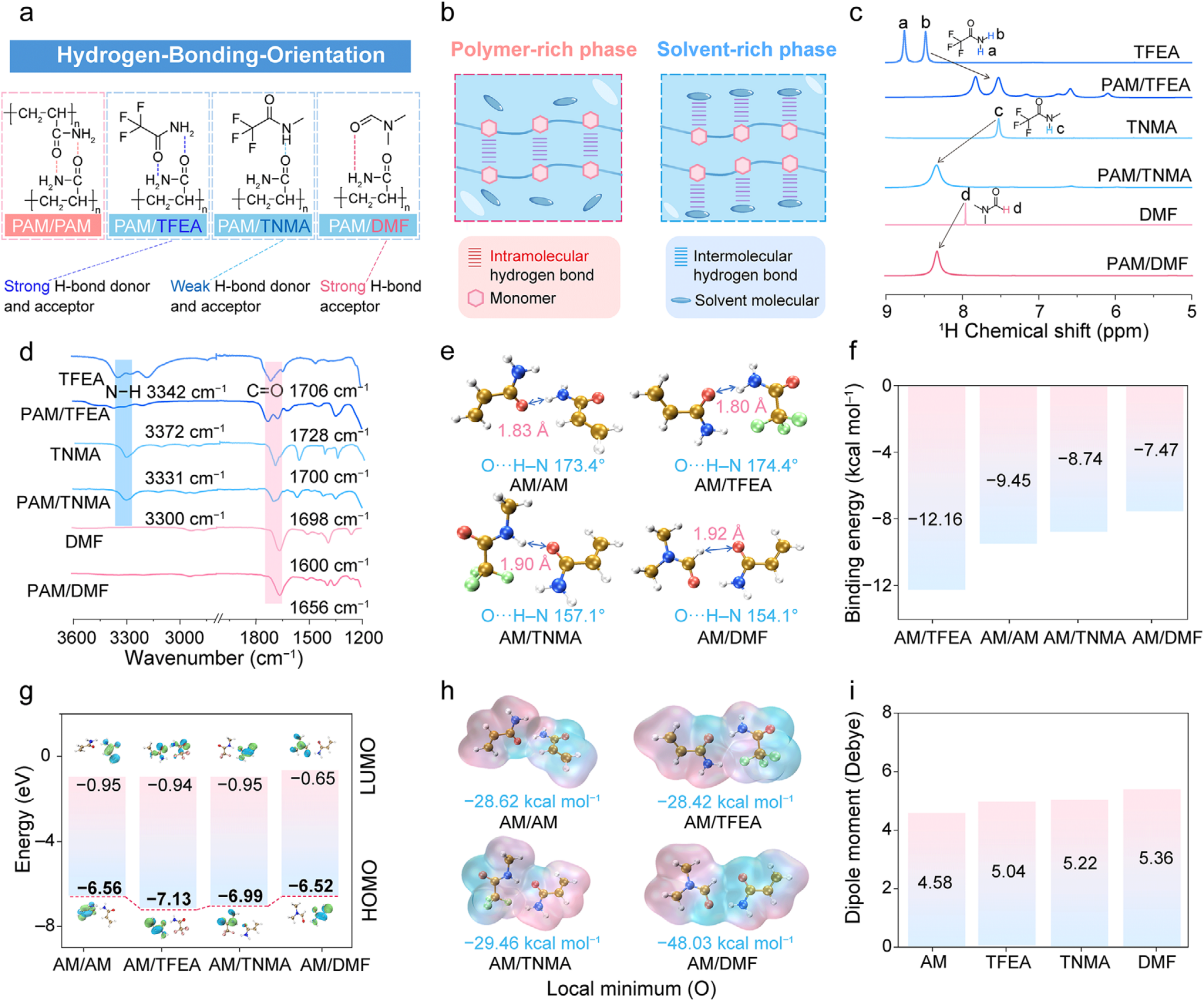
**Hydrogen‐bonding‐interaction bicontinuous phase separation mechanism**. (a) Schematic of the interactions between different types of H‐bonds and (b) corresponding intramolecular (polymer‐polymer) hydrogen bond in the polymer‐rich phase and intermolecular (polymer‐solvent) hydrogen bond in the asolvent‐rich phase. (c) ^1^H NMR and (d) FTIR spectra for PAM‐based DEGs prepared in solvents with varying hydrogen bond donor strengths (TFEA > TNMA > DMF). (e) DFT‐optimized geometries and (f) calculated hydrogen‐binding energies for AM‐AM self‐association versus AM‐solvent interactions. (g) HOMO‐LUMO energy diagrams and (h) calculated electrostatic potential (ESP) maps illustrating the electronic basis for the interaction strengths. (i) A comparison of molecular dipole moments, revealing that local N─H bond polarity, not the global dipole moment, governs the hydrogen bond donor strength and drives the phase separation.

To unravel the molecular origins of this spontaneous phase separation in DEG systems, we investigated the role of hydrogen bond competition in controlling polymer network assembly in simpler model systems. As conceptualized in Figure 3a, the PAM chain can form strong intramolecular hydrogen bonds with itself (PAM/PAM). This self‐association is challenged by the intermolecular hydrogen bonding offered by the solvents. The outcome of this competition, depicted in Figure 3b, dictates the final network morphology: dominant PAM‐PAM interactions drive phase separation to form a polymer‐rich phase, whereas effective PAM‐Solvent interactions lead to a homogeneous, solvent‐rich gel. To experimentally validate this hypothesis, we systematically modulated this competition by polymerizing acrylamide in three distinct solvent environments, each selected for its systematically varying hydrogen bond donor strength: (i) trifluoroacetamide (TFEA), which has a primary amino group (─NH_2_) and acts as a strong hydrogen bond donor, (ii) *N*‐methyl‐2,2,2‐trifluoroacetamide (TNMA), with a secondary amide (─NH─) as an intermediate donor, and (iii) dimethylformamide (DMF), which is a pure acceptor and lacks donor protons. Notably, DMF was not chosen as a structural analogue of TNMA, but as an acceptor‐only control to define the mechanistic boundary of the proposed competitive hydrogen‐bonding framework. Its lack of an N─H donor allows us to evaluate whether acceptor‐type interactions alone can sufficiently weaken AM‐AM self‐association. This design yielded three comparative DEGs (PAM/TFEA, PAM/TNMA, and PAM/DMF), allowing us to isolate and directly observe how the solvent's donating ability controls the competition between polymer‐polymer and polymer‐solvent interactions and the resulting phase behaviour.

Nuclear magnetic resonance (^1^H NMR) and FTIR spectroscopy of these DEGs reveal distinct hydrogen‐bonding landscapes that correlate with their macroscopic phase behaviour. In the PAM/TFEA DEG, the N─H proton signal shifted from δ 8.74 to 7.83 ppm, indicating enhanced proton shielding. Simultaneously, the C═O stretching vibration underwent a notable blue shift from 1706 to 1728 cm^−1^, along with broadening of the N─H band at 3342 cm^−1^. These changes are consistent with the formation of strong intermolecular N─H···O═C hydrogen bonds between TFEA and AM, which disrupt PAM's intrinsic self‐association. Macroscopically, the PAM/TFEA DEG is transparent and uniform (similar to the PDMA‐rich AD0 DEG), indicating minimal phase separation because the strong donor TFEA kept the PAM chains well solvated and apart from each other (Figure S15). In contrast, the TNMA‐ and DMF‐based gels show downfield shifts to 8.33 ppm (from 7.51 and 7.95 ppm, respectively) in the N─H proton signal, indicating enhanced deshielding effects (Figure 3c). This shift corresponds to subdued donor competition, which permits polymerization to entrench PAM‐PAM interactions, thereby depleting N─H electron clouds. Minimal FTIR shifts (C═O and N─H bands) in these systems affirm the dominance of polymer‐centric interactions (Figure 3d). Visually, both TNMA‐ and DMF‐based PAM DEGs are opaque and showed obvious phase separation or precipitation of polymer‐rich regions (Figure S15), directly tying the lack of strong solvent competition to poor polymer–solvent compatibility and network heterogeneity.

This competition‐driven assembly profoundly influences the bulk material properties. X‐ray diffraction (XRD) patterns of the PAM/TFEA DEG showed distinct crystalline peaks, indicative of a highly ordered, long‐range structure templated by uniform intermolecular hydrogen bonding. Conversely, the PAM/TNMA and PAM/DMF DEGs were amorphous, consistent with the disordered networks formed through phase separation (Figure S16). This structural dichotomy is reflected in the thermal and mechanical properties. The PAM/TFEA DEG possesses the highest glass transition temperature (T_g_) of −60.68°C, indicating less polymer chain mobility due to solvent‐polymer interactions. In contrast, TNMA‐ and DMF‐based DEGs have lower T_g_ (−87.39°C and −91.16 °C, respectively), reflecting increased segmental freedom when polymer chains cluster together and solvent is excluded (Figure S17). Mechanically, while the uniform PAM/TFEA network enabled excellent stretchability (473%) at modest strength (0.028 MPa), the heterogeneous PAM/TNMA and PAM/DMF gels are brittle with limited extensibility due to stress concentration at aggregated polymer‐rich domains (Figure S18).

To further probe the competitive‐hydrogen‐bonding‐regulated phase‐separation mechanism, 2D ^1^H═^1^H nuclear Overhauser effect spectroscopy (NOESY) NMR, variable‐temperature Fourier transform infrared (VT‐FTIR) spectroscopy, and two‐dimensional correlation spectroscopy (2D‐COS) analyses were performed (Figures S19–S21). The NOESY spectra indicate distinct local interaction patterns in PAM/TFEA and PAM/TNMA, while the VT‐FTIR and 2D‐COS results show that PAM/TFEA undergoes more pronounced perturbation of its N═H─ and C═O‐related interaction environment than PAM/TNMA. These observations suggest that TFEA more effectively disrupts PAM‐rich self‐association, whereas TNMA preserves it to a greater extent, consistent with the phase‐behavior differences observed experimentally.

To provide a first‐principles basis for these experimental findings, we performed density functional theory (DFT) calculations to quantify the interaction energetics, geometries, and electronic structures [34]. As the donor strength of the solvent decreases from TFEA to TNMA to DMF, the O···H─N bond lengths progressively elongate (1.80 Å to 1.90 Å to 1.92 Å), and the bond angles deviate from ideal linearity (173.4° to 157.1° to 154.1°) (Figure 3e). These changes weaken the hydrogen bond stability, as optimal hydrogen bonds favor nearly linear alignments for maximal orbital overlap. Interaction energies follow a similar trend, with AM/TFEA exhibiting the strongest interaction (−12.16 kcal mol^−1^), surpassing the AM‐AM interaction energy (−9.45 kcal mol^−1^) and leading to the disruption of intrachain bonds. In comparison, TNMA (−8.74 kcal mol^−1^) and DMF (−7.47 kcal mol^−1^) interactions are weaker, favoring PAM chain aggregation and phase separation (Figure 3f). These energetic differences are accompanied by changes in the frontier molecular orbital energy levels. Upon solvent–polymer complex formation, electron‐density redistribution within the donor–acceptor pair reduces the electron‐donating character of the complex, which is reflected by the lowered HOMO energy levels (Figures 3g and S22) [35]. This HOMO lowering indicates electronic‐structure modulation and enhanced oxidative stability after complex formation. Particularly, the more negative electrostatic potential of AM/TFEA (s28.42 kcal mol^−1^) reflects a heightened attraction between the donor site and polymer, reinforcing electrostatic‐driven bond formation (Figures 3h and S23). RDG analysis and differential electron density plots further support the proposed interaction mechanism (Figures S24 and S25). These results confirm attractive weak noncovalent interactions in the AM‐containing complexes and reveal distinct charge‐redistribution patterns, supporting the directional hydrogen‐bond‐type interactions of TFEA/TNMA with AM.

Strikingly, the superior hydrogen‐bonding capability of TFEA appears paradoxical when considering the global molecular dipole moments, which follow the order DMF (5.36 D) > TNMA (5.22 D) > TFEA (5.04 D). This inverse correlation reveals a crucial molecular design principle: hydrogen bond donor strength is dictated not by global molecular polarity, but by the local electronic environment and steric accessibility of the donor N─H group. While methyl substitution in TNMA and DMF increases the overall dipole moment, it concurrently diminishes the N─H group's local polarity and introduces steric hindrance, crippling its ability to act as an effective hydrogen bond donor. This fundamental insight validates our hypothesis and provides a clear rationale for the molecular competition that drives the formation of our functional, phase‐separated electrolyte architecture (Figure 3i).

### 2.3 Mechanical Properties and Energy Dissipation Mechanisms

**FIGURE 4:**
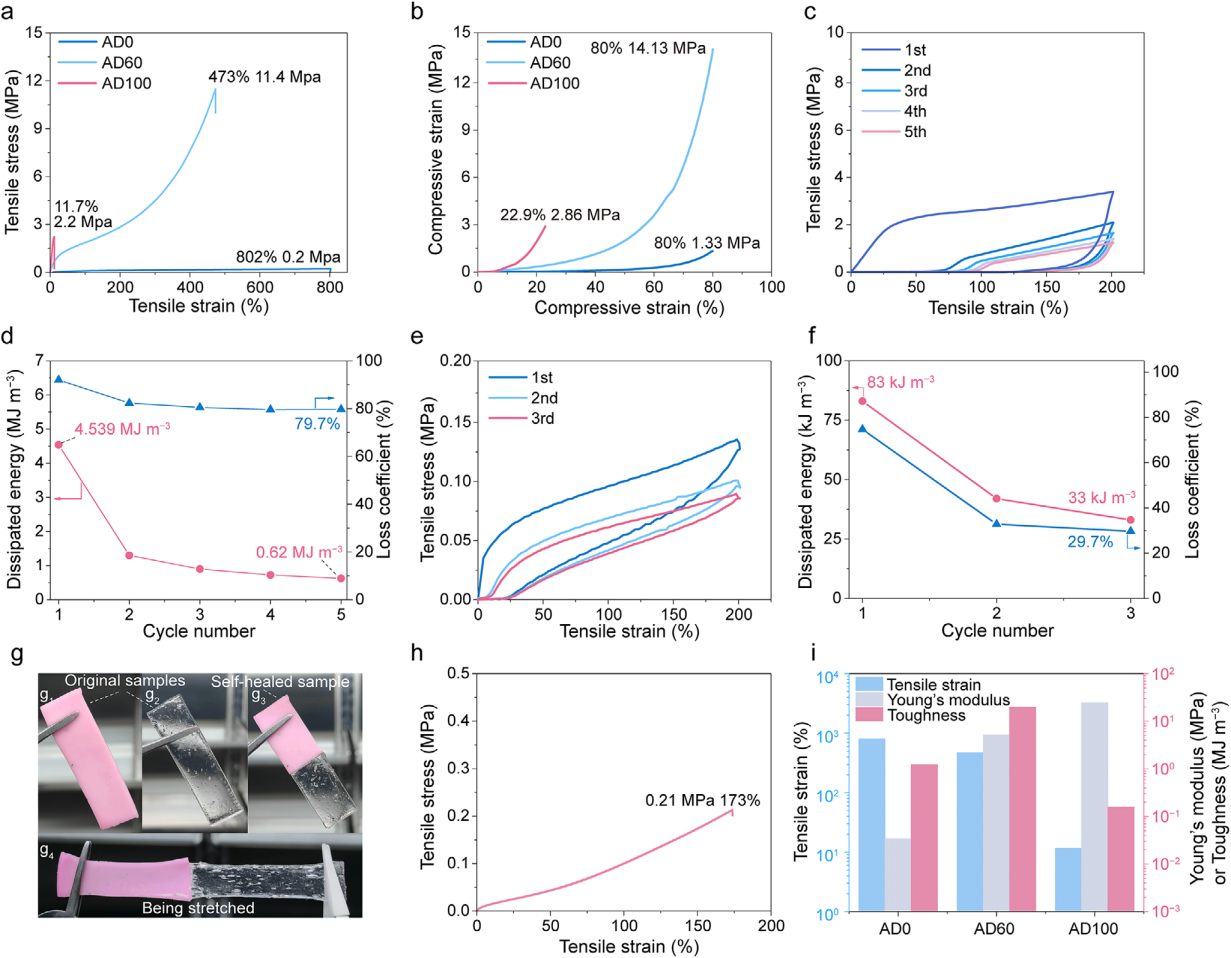
Mechanical performance and energy dissipation of the bicontinuous DEG. (a) Tensile and (b) compressive stress‐strain curves for the AD0, AD60, and AD100 samples. (c) Cyclic tensile loading curves for AD60 under 200% strain and (d) its corresponding energy dissipation for each cycle. (e) Cyclic tensile loading curves for AD0 under 200% strain and (f) its corresponding energy dissipation for each cycle. (g) Digital images of the original DEG samples before stretching in pink color (g_1_) and in transparent form (g_2_), the self‐healed DEG sample (pink section is seamlessly connected with transparent section) (g_3_), and the self‐healed sample being stretched (g_4_). (h) The tensile stress‐strain curve of the self‐healed electrolyte sample. (i) The comparison of the AD samples in terms of toughness, Young's modulus, and tensile strength.

The engineered phase‐separated architecture endows the DEG electrolytes with a remarkable combination of strength and toughness, far exceeding that of conventional single‐phase gels. To quantify these advantages, we carried out comprehensive mechanical testing on three representative formulations: AD0 (pure PDMA, no phase separation), AD100 (pure PAM, extreme phase separation/aggregation), and AD60 (optimized bicontinuous network). Tensile testing revealed a remarkable synergy between the soft PDMA‐rich and hard PAM‐rich phases (Figure 4a). The AD0 sample, lacking reinforcing motifs, exhibits hyper‐elasticity with a strain of over 800%, but only achieves a minimal tensile strength of 0.2 MPa. This is due to unrestrained chain slippage, which dissipates stress inefficiently. In contrast, the AD100 sample, with a dense PAM hydrogen bond network, exhibits high stiffness but intrinsic brittleness, suffering from catastrophic failure at only 11.7% strain. Its stress–strain curve showed a typical brittle fracture behavior with virtually no yielding, attributable to the dense hydrogen‐bonded PAM network that, without soft segments, cannot accommodate strain. Remarkably, the AD60 sample, which incorporates a balanced ratio of PAM and PDMA, shows a bicontinuous microstructure, combining both strength (11.4 MPa) and stretchability (473%). Its curve showed an initial elastic regime followed by pronounced strain hardening, indicating that as stress increased, more polymer chains were recruited to bear load. In this bicontinuous structure, the hard PAM aggregates act as sacrificial nodes, bearing load and allowing reversible fracture, while the soft PDMA channels absorb deformation via entropic recoil. This dual mechanism creates a toughening effect reminiscent of biological materials like spider silk, where crystalline domains provide strength while amorphous regions enable extensibility [36, 37]. The resilience induced by phase separation extends beyond tensile loading to compressive loads, where AD60 endures up to 14.13 MPa at 80% strain, compared to AD0 and AD100, which show much lower resistance to deformation (Figure 4b).

Cyclic loading tests further explored the energy dissipation kinetics. AD60 exhibited pronounced hysteresis loops at 200% strain, with an initial dissipation of 4.539 MJ m^−3^, which stabilized at 0.62 MJ m^−3^ during post‐cycling. The loss coefficient stabilized at 79.7%, dropping from 92.01% after the first cycle, indicating significant energy dissipation without macroscopic damage (Figure 4c,d). This resilience arises from the fracture of interchain hydrogen bonds during deformation, allowing sustained energy dissipation as heat, while the interlocked topology ensures reformation and cycle invariance [38]. In contrast, AD0, which lacks such mechanisms, exhibits minimal dissipation (83 kJ·m^−3^ initially, decaying to 33 kJ·m^−3^), with a rapid decrease in the loss coefficient from 74.7% to 29.7%, as viscous damping alone leads to irreversible entanglement losses (Figure 4e,f). Pure PAM DEG, being too brittle, could not undergo this testing.

The phase‐separated DEG also exhibits remarkable self‐recovery and self‐healing properties. After high‐strain loading (400% strain), the AD60 DEG retains some deformation but recovers nearly its original dimensions after resting at ambient temperature for several minutes (Figure S26 and Video S3). This self‐recovery is attributed to the gradual reformation of disrupted hydrogen bonds after unloading. More notably, these DEGs exhibit self‐healing capabilities after mechanical damage (Figure 4g,h). When two differently colored gel fragments are placed in contact at 60°C for just one minute, effective healing is achieved, and the repaired sample withstands over 173% strain before fracture, with a fracture strength of approximately 0.21 MPa. Although the post‐healing strength is slightly lower than the initial value, this demonstrates that hydrogen bond networks can rapidly re‐establish connections under thermal activation, effectively repairing fractures. This characteristic is of particular significance for battery safety, as microcracks in the DEG electrolyte can be repaired, maintaining contact between the electrode and electrolyte and preventing performance degradation [39]. In addition, Young's modulus distributions shift progressively with AM content: AD0 (0.034 MPa), AD60 (5.14 MPa), and AD100 (24.39 MPa). Crucially, the AD60 sample achieves the highest toughness of 19.91 MJ m^−^^3^, significantly outperforming both the soft AD0 and the brittle AD100. The steep rise in stiffness with increasing AM content is directly correlated with the enhanced formation of hydrogen‐bonded clusters, confirming the structural reinforcement role of these interactions (Figure 4i).

Through this integrated mechanistic and experimental analysis, we have developed a comprehensive structure‐property relationship framework for the phase‐separated DEGs. AM‐deficient networks form soft, extensible but mechanically compromised matrices due to minimal interchain interactions, while networks with excessive AM content (AD100) become overly rigid and brittle with high modulus but limited deformability. In phase‐separated DEGs, however, these characteristics complement each other: rigid domains provide structural reinforcement, while soft domains maintain matrix flexibility. The phase‐separated architecture imparts exceptional fracture toughness to the DEG, arising from dynamic, reversible sacrificial bonds within the polymer‐rich regions. When the DEG experiences tensile or compressive loads, hydrogen bond networks within polymer‐rich domains preferentially stretch and rupture, absorbing energy; upon unloading, these noncovalent bonds can reform, enabling partial structural recovery. This process parallels the toughening principle found in double‐network hydrogels, where one network functions as a “sacrificial” energy‐dissipating component. In high‐solvent‐content gels, a trade‐off typically exists between rigidity and extensibility. Our system demonstrates that the precise balance between hydrogen‐bonding (which induces phase separation) and solvent compatibility (which preserves network flexibility) is crucial to optimizing mechanical performance in DEG electrolytes.

### 2.4 Ion Transport in the Phase‐Separated Electrolyte

**FIGURE 5:**
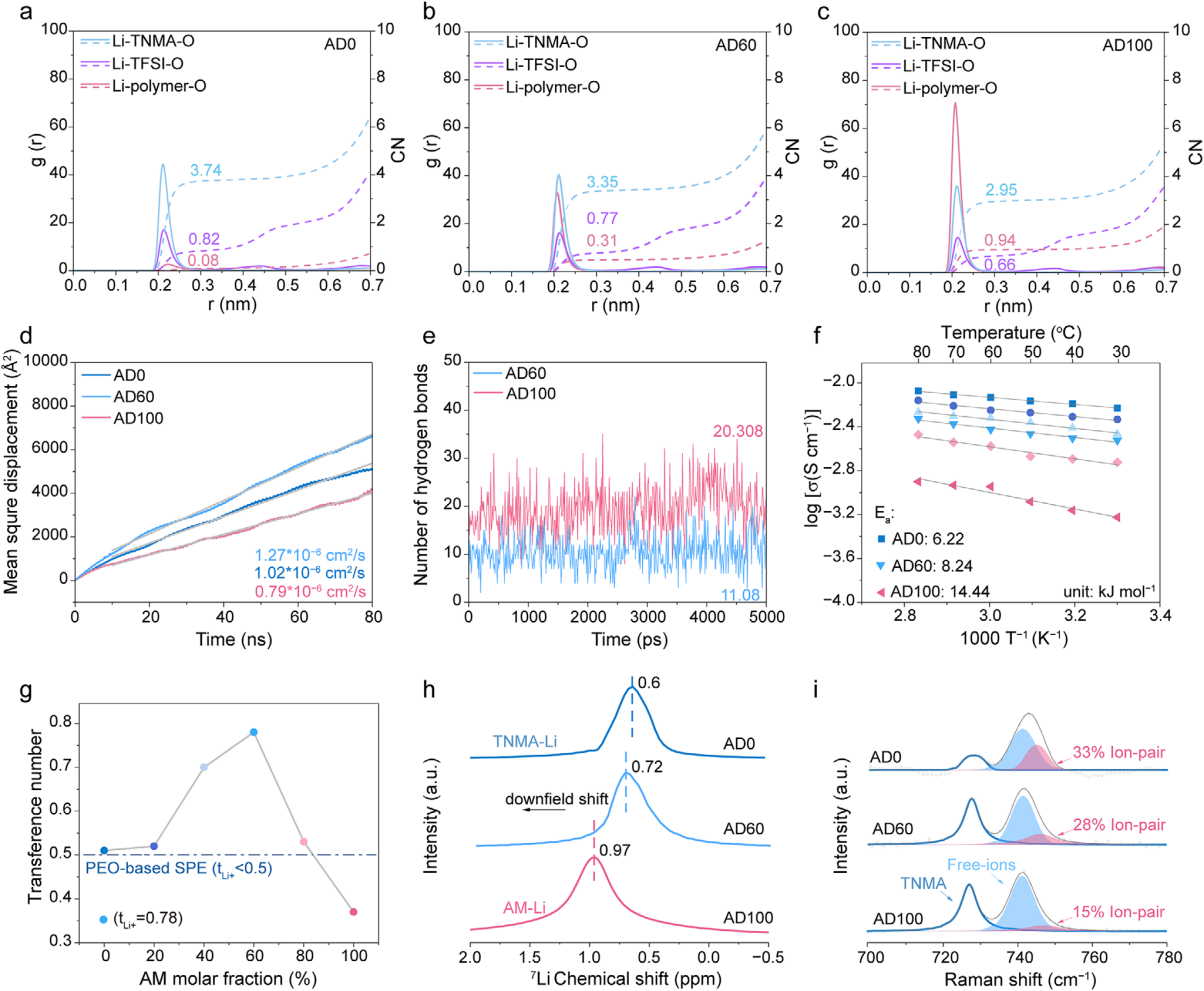
**Evaluation of the ion transport of the bicontinuous DEG**. Radial distribution functions (RDFs) and coordination numbers (CN) of Li^+^ interacting with solvent, polymer, and anions in (a) AD0, (b) AD60, and (c) AD100. (d) Mean square displacement (MSD) of Li^+^ in AD0, AD60, and AD100 DEGs. (e) Hydrogen bond statistics between polymer chains from MD simulations in AD60 and AD100 (f) Vogel‐Tammann‐Fulcher plots of ionic conductivity for DEGs with varying AM content. The AD60 formulation maintains a high conductivity of 2.99 mS cm^−^^1^ at 30°C. (g) Li^+^ transference number ($t_{Li^{+}}$) as a function of AM molar fraction, peaking at an exceptional value of 0.78 for AD60. (h) ^7^Li solid‐state NMR, and (i) Raman spectra of AD0, AD60, and AD100 DEGs.

To elucidate the microscopic mechanism of decoupled ion transport, the intermolecular interactions and solvation structures within the electrolyte were investigated via classical molecular dynamics (MD). Figures 5a–c and S27 illustrate the variations in the radial distribution function (RDF) and coordination number (CN) in different DEGs. As shown in Figure S27, the RDF between the polymer skeleton and TFSI^−^ in AD60 exhibits a distinct coordination peak at ∼0.32 nm, whereas no such characteristic peak is observed in AD0. The coordination numbers (CNs) of Li^+^ with TNMA and TFSI^−^ decrease from the values in AD0 (3.74 and 0.82) to the lowest levels in AD100 (2.95 and 0.66) (Figure 5a,c). Notably, AD60 maintains an optimal intermediate configuration (3.35 and 0.77), which facilitates a synergistic balance between anion‐anchoring and rapid ion release (Figure 5b). This specific configuration implies that Li^+^ migrates with a streamlined solvation sheath, thereby minimizing steric hindrance to facilitate rapid ion transport. Figure 5d presents the mean squared displacement (MSD) profiles, where AD60 achieves a remarkable Li^+^ diffusion coefficient of 1.27 × 10^−6^ cm^2^ s^−1^. This value surpasses that of the homogeneous AD0 (1.02 × 10^−6^ cm^2^ s^−1^) and is higher than the aggregated AD100 (0.79 × 10^−8^ cm^2^ s^−1^), which is hindered by the strong polymer‐ion trapping effect. This accelerated transport is thermodynamically rationalized by the interaction energy evolution (Figure S28), which reveals that Li^+^ in AD60 experiences the weakest electrostatic binding to anions (least negative energy) and negligible interaction with the polymer skeleton. Figure 5e shows that the average number of inter‐polymer H─bonds nearly doubles from 11.08 in AD60 to 20.308 in AD100, confirming an ultra‐dense self‐associating framework. The ionic conductivity of the DEG electrolytes was measured across a range of temperatures to explore the temperature‐dependent transport behaviour. All samples follow typical Vogel‐Tammann‐Fulcher (VTF) behaviour, consistent with polymer segment–assisted dynamics superimposed on liquid‐like motion in the DES‐rich phase (Figures 5f and S29). The pure PDMA (AD0) exhibits a respectable conductivity (5.90 mS cm^−1^) at 30°C (Figure S30), which confirms that a continuous solvent network persists when strong hydrogen bond donors are absent. Adding AM gradually lowers conductivity as PAM‐rich domains grow and narrow the solvent pathways. Beyond the optimum the decline becomes sharp, from 2.99 mS cm^−1^ in AD60 to 1.89 mS cm^−1^ in AD80 and 0.595 mS cm^−1^ in AD100 at 30°C, which signals percolation of rigid PAM networks that interrupt the ion transport via DES domains. In parallel, the apparent activation energy obtained from VTF fits increases monotonically with AM content, with values near 5.02 kJ mol^−1^ for the DES, 8.24 kJ mol^−1^ for AD60 and 14.44 kJ mol^−1^ for AD100 (Figures 5f and S31). This trend indicates a shift from low‐barrier solvent‐mediated hopping to higher‐barrier transport assisted by polymer segmental motion as PAM becomes dominant. Furthermore, the glass transition temperature (T_g_) decreases from −80.0°C in the homogeneous AD0 to −86.3°C in AD60 further reflects the formation of a highly mobile solvent‐rich phase induced by phase separation (Figure S32).

The selectivity of ionic transport, captured by the lithium‐ion transference number ($t_{Li^{+}}$), shows a more pronounced and nonmonotonic composition dependence (Figures 5g and S33). As the AM fraction increases, the $t_{Li^{+}}$ first rises from a moderate 0.51 in AD0 to a remarkable peak of 0.78 in AD60, which reveals that the bicontinuous morphology maintains rapid lithium motion in PDMA/DES channels while suppressing anion mobility. Beyond this optimum, however, the $t_{Li^{+}}$ drops to 0.37 in AD100. This loss arises from over‐coordination of lithium by carbonyl sites on PAM, which restricts cation mobility and disrupts the continuity of conductive pathways [40]. These findings suggest that a balanced phase separation is critical to enhancing the selective transport of lithium ions without imposing a prohibitive transport barrier. Therefore, the high $t_{Li^{+}}$ of AD60 arises from the balanced interplay among anion tethering, moderate Li^+^ coordination, and ion‐channel mobility enabled by optimized PAM incorporation, rather than from the effect of a single component.

Density functional theory (DFT) calculations (Figures S34–S36) reveal that at low concentrations, the N─H groups in AM may form strong interactions with TFSI^−^ anions (binding energy: −20.02 kcal mol^−1^), effectively immobilizing anions and enhancing the Li^+^ transference number. At high concentrations, however, although AM's binding affinity for Li^+^ (−10.58 kcal mol^−1^) is weaker than that of N‐methyl‐2,2,2‐trifluoroacetamide (TNMA, −18.39 kcal mol^−^^1^) (TNMA, −18.39 kcal mol^−1^), excess AM competes with TNMA for Li^+^ coordination. This competition introduces inefficient Li^+^ hopping pathways, impairing ionic conductivity and disrupting continuous transport. In contrast, DMA (lacking donors) interacts weakly with anions and preserves a DES‐like solvation environment. Although DMA/PDMA also contains C═O groups that can coordinate with Li^+^, the weaker DMA–Li^+^ binding energy and the MD‐derived coordination numbers indicate that PDMA C═O sites contribute only weakly to the Li^+^ coordination environment. This analysis establishes the dual role of the PAM component: it can immobilize anions via N─H···TFSI^−^ interactions to raise $t_{Li^{+}}$, but its C═O sites also strongly coordinate Li^+^, which can suppress overall mobility if the PAM content is too high. Complementarily, electrostatic potential (ESP) analysis (Figure S36) assesses each monomer's anion‐adsorption capacity. The ESP max value, indicative of the ability to form H─bonds with anions, is lowest for DMA (30.5 kcal mol^−1^) and higher for AM (53.6 kcal mol^−1^), confirming that AM interacts strongly with anions and significantly perturbs the solvation structure.

Spectroscopic analysis provides direct experimental validation of this competitive landscape. FTIR shows a progressive blue shift of the C═O stretch from 1704 cm^−1^ (AD0) to 1708 cm^−1^ (AD100), consistent with increasing Li^+^ coordination to PAM carbonyls, while the N─H stretch shifts from 3305 to 3309 cm^−1^ and broadens, evidencing stronger N─H···TFSI^−^ hydrogen bonding (Figure S37). To further probe the coupling between PAM amide (N─H) groups and TFSI^−^ anions, synchronous 2D‐COS maps were generated from variable‐temperature FTIR spectra (Figure S38). The analysis focused on the PAM ν(N─H) region (3600–3200 cm^−^^1^) and the TFSI^−^ ν_as_(SO_2_) region (1340–1370 cm^−^^1^). Compared with AD60, AD100 exhibited a stronger positive correlation between these two regions, suggesting enhanced coupling between PAM N─H and TFSI^−^ SO_2_ vibrations at higher PAM content. This correlated response is consistent with enhanced coupling between PAM N─H group and TFSI^−^ anions, suggesting the presence of local PAM–TFSI^−^ interactions. As the PAM content increases, the Li^+^ coordination environment progressively shifts. This is corroborated by solid‐state ^7^Li NMR (Figure 5h), where a progressive downfield shift of the resonance from 0.6 ppm (AD0) to 0.97 ppm (AD100) confirms that Li^+^ nuclei are transitioning to the more electron‐withdrawing environment of the PAM carbonyl groups. On another front, we tracked the state of the TFSI^−^ anion via Raman spectroscopy (Figure 5i). As shown in Figure 5i, the fraction of the Ion‐pair species exhibits a systematic decrease from 33% in AD0 to 28% in AD60, and further to 15% in AD100 with increasing AM content. In the optimal AD60, the bicontinuous morphology enables functional decoupling. The flexible, DES‐rich PDMA phase forms continuous ion highways where Li^+^ migration occurs via a low‐energy, solvent‐mediated hopping mechanism. In parallel, the rigid PAM‐rich phase acts as a highly effective anion trap. The dense hydrogen‐bonding network immobilizes TFSI^−^ anions into a stationary polymer‐bound state, thereby suppressing anion mobility without hindering cation transport. This efficient anion immobilization is the direct cause of the exceptionally high transference number. As the AM content increases beyond the optimum, however, the PAM‐rich domains percolate and disrupt these highways. The transport mechanism is consequently forced into a high‐energy, polymer segmental motion‐assisted pathway. In this regime, the overabundant PAM network not only traps anions but also excessively coordinates Li^+^, severely impeding all ion movement and causing a collapse in both conductivity and the transference number. Finally, this optimized solvation structure also enhances electrochemical stability (Figure S39). Linear sweep voltammetry reveals that the AD60 and AD100 gels are stable up to ∼4.7 V vs Li^+^/Li, a significant improvement over the ∼4.0 V for the liquid DES. The coordination of solvent and anions by the polymer network reduces their activity at the electrode interface, suppressing oxidative decomposition and making the DEGs suitable for high‐voltage battery applications.

### 2.5 Interface Engineering and Dendrite Suppression

**FIGURE 6:**
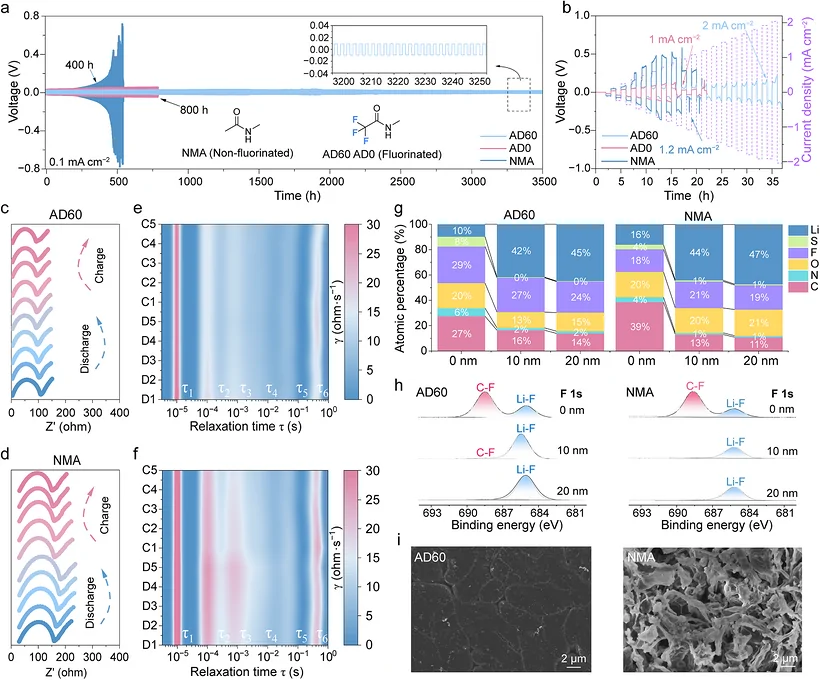
**Dendrite suppression in Li||Li symmetric cells**. (a) Galvanostatic Li plating and stripping profiles of the Li|DEG|Li symmetric cell at 0.1 mA cm^−2^, 0.1 mAh cm^−2^. (b) Critical current density (CCD) of the Li||Li symmetric cells at various current densities from 0.1 to 2.0 mA cm^−2^. (c, d) In situ EIS Nyquist plots and (e, f) corresponding distribution of relaxation times (DRT) analysis during cycling. (g) Atomic percentages and (h) high‐resolution F 1*s* XPS spectra from the lithium anode surface after cycling, confirming the formation of a stable and LiF‐rich SEI in the AD60 system. (i) SEM images of lithium metal anode after cycling of Li|DEG|Li symmetric cell for 300 h at 0.1 mA cm^−2^.

We selected TNMA as the fluorinated amide component to regulate the Li‐metal interface through coupled electronic, solvation, and network‐environment effects. Relative to its nonfluorinated analogue N‐methylacetamide (NMA), TNMA exhibits a lower LUMO energy level, indicating greater participation in reductive interfacial chemistry (Figures S40 and S41). Fluorination also weakens Li^+^‐amide coordination and promotes greater TFSI^−^ participation in the primary Li^+^ solvation shell, which is favorable for anion‐involved inorganic interphase formation (Figures 5b and S42). In addition, after polymerization, the DEG forms a highly salt‐rich, “salt‐shielded” polymer environment in which the amide‐containing components are constrained by hydrogen‐bonding interactions among PAM, TNMA, and TFSI^−^, thereby reducing the mobility and direct interfacial exposure of reactive N─H ‐containing sites to Li metal (Figure S43). These features provide the design basis for a more stable Li‐metal interface and for the formation of a LiF‐rich interphase through fluorinated‐solvent‐ and anion‐involved interfacial reactions. To validate this strategy, we performed long‐term galvanostatic cycling of Li|DEG|Li symmetric cells and compared the performance of our optimized AD60 with several controls: a nonfluorinated analogue using N‐methylacetamide (NMA) and the mechanically weak AD0 DEG. As shown in Figure 6a, theAD60 electrolyte demonstrated exceptional cycling stability and maintained a stable voltage profile for over 3500 hours under 0.1 mA cm^−2^ and 0.1 mAh cm^−2^. In contrast, the AD0 electrolyte failed catastrophically after approximately 800 hours, likely due to insufficient mechanical strength to suppress lithium dendrite growth. The NMA‐based cell suffered from progressive voltage hysteresis and premature short‐circuiting within 600 hours, indicative of unstable SEI formation and growing interfacial impedance. This phenomenon is more pronounced at high current densities (0.2 mA cm^−2^/0.2 mAh cm^−2^), where the AD0 electrolyte without phase separation short‐circuits around 500 h due to uncontrolled lithium dendrite growth, whereas AD60 sustains a low overpotential (30 mV) and stable cycling over 3500 h (Figure S44). This pronounced difference highlights the superior efficacy of the phase‐separated AD60 structure in suppressing lithium dendrite formation. Even at a high current density of 0.5 mAh, the AD60 electrode maintains stable cycling for up to 1000 h (Figure S45). The critical current density (CCD) was determined by stepwise increasing the current density from 0.1 to 2.0 mA cm^−2^ (Figure 6b). The polarization voltage profiles of the AD60 cell increased with rising current densities, but the cell continued stable cycling even at 2.0 mA cm^−2^. In contrast, the AD0 and NMA‐based cells suffered from short circuits at lower current densities of 1 mA cm^−2^ and 1.2 mA cm^−2^, respectively. These results demonstrate that the phase‐separated TNMA‐containing DEG delivers exceptional interfacial stability and dendrite suppression, far beyond the nonfluorinated analog and vastly better than a typical GPE.

In situ electrochemical impedance spectroscopy (EIS) was performed to track the evolution of interfacial kinetics during Li stripping/plating cycling. As shown in Figure 6c,d, the TNMA‐based DEG demonstrated consistently lower impedance compared to the NMA‐based DEG, suggesting the formation of a more stable SEI layer with enhanced ion transport kinetics. Distribution of relaxation time (DRT) analysis was performed to deconvolute the contact resistance (R_c_), interfacial resistance (*R*_SEI_), and the charge transfer resistance (R_ct_) from the Nyquist plots (Figure 6e,f). It can be observed that the feature at τ_1_ ∼10^−5^ s corresponds to the R_c_, which is relatively small for both gel electrolytes and shows negligible differences. The NMA system exhibits pronounced, high‐intensity peaks (τ_2_ and τ_3_) in this region, indicative of substantial interfacial resistance (R_SEI_). Conversely, these features are effectively suppressed in the AD60 system, manifesting as a low‐intensity distribution that corroborates the significantly reduced interfacial impedance observed in the corresponding Nyquist plots. The low‐frequency peak at τ_6_ ∼10^0^ s corresponds to the mass transport of Li^+^ ions, reflecting the diffusion process within the gel electrolyte. While this feature remains highly stable in the AD60 system, the NMA system exhibits significant fluctuations in peak intensity and position, indicating an unstable diffusion interface caused by uneven lithium deposition.

The elemental ratios (Figure 6g) indicate a robust fluorinated interface for AD60, where a high F content of ∼24.5–28.9% is preserved from the surface down to 20 nm. The F 1s spectra (Figure 6h) reveal that the LiF component at 684.95 eV accounts for a higher relative proportion in AD60 (31.7%) compared to NMA (24.9%). Furthermore, Li_2_CO_3_ in the SEI has been associated with dendrite formation. As shown in Figure S46, AD60 displays a weaker Li_2_CO_3_ peak at 531.2 eV. Meanwhile, AD60 exhibits a higher contribution of Li_2_O (33.7%) compared to 27.0% in NMA at 20 nm. The fluorinated groups in AD60 facilitate coordination of the amide nitrogen atoms with Li^+^, leading to their reduction to Li*_x_* N during electrochemical cycling, with the signal intensifying upon deeper etching. For the inorganic components, the SEI in AD60 has discernible quantities of Li_2_O, LiF, Li*_x_* N (Figure S47), encapsulated within the F‐rich DEG. These inorganic constituents act as reinforcing agents, effectively suppressing Li dendrite growth.

This chemically distinct SEI architecture has profound consequences for the lithium deposition morphology (Figures 6i and S48). In the AD60 electrolyte, the dense, inorganic‐rich SEI promotes uniform and compact lithium deposition, effectively suppressing the formation of porous, high‐surface‐area structures and inactive “dead” lithium. This ideal morphology is the direct result of the SEI's mechanical robustness and efficient Li^+^ transport. Conversely, the unstable, organic‐rich SEI in the NMA electrolyte fails to regulate ion flux, leading to the growth of abundant dendrites and porous, sponge‐like lithium, which correlates with its rapid performance decay and rising interfacial resistance. Notably, this design logic differs from that of recent fluorinated amide‐based DEG studies that mainly emphasize molecular screening for Li‐metal interfacial chemistry [30]. Here, the fluorinated amide component is not used solely for interfacial optimization, but also as part of a competitive hydrogen‐bonding environment that governs DEG‐network self‐organization and nanoscale phase separation. This dual role extends fluorinated DEG design from molecular/interfacial tuning to programmable gel‐network structuring.

### 2.6 Performance of Solid‐State Li‐Metal Batteries

**FIGURE 7:**
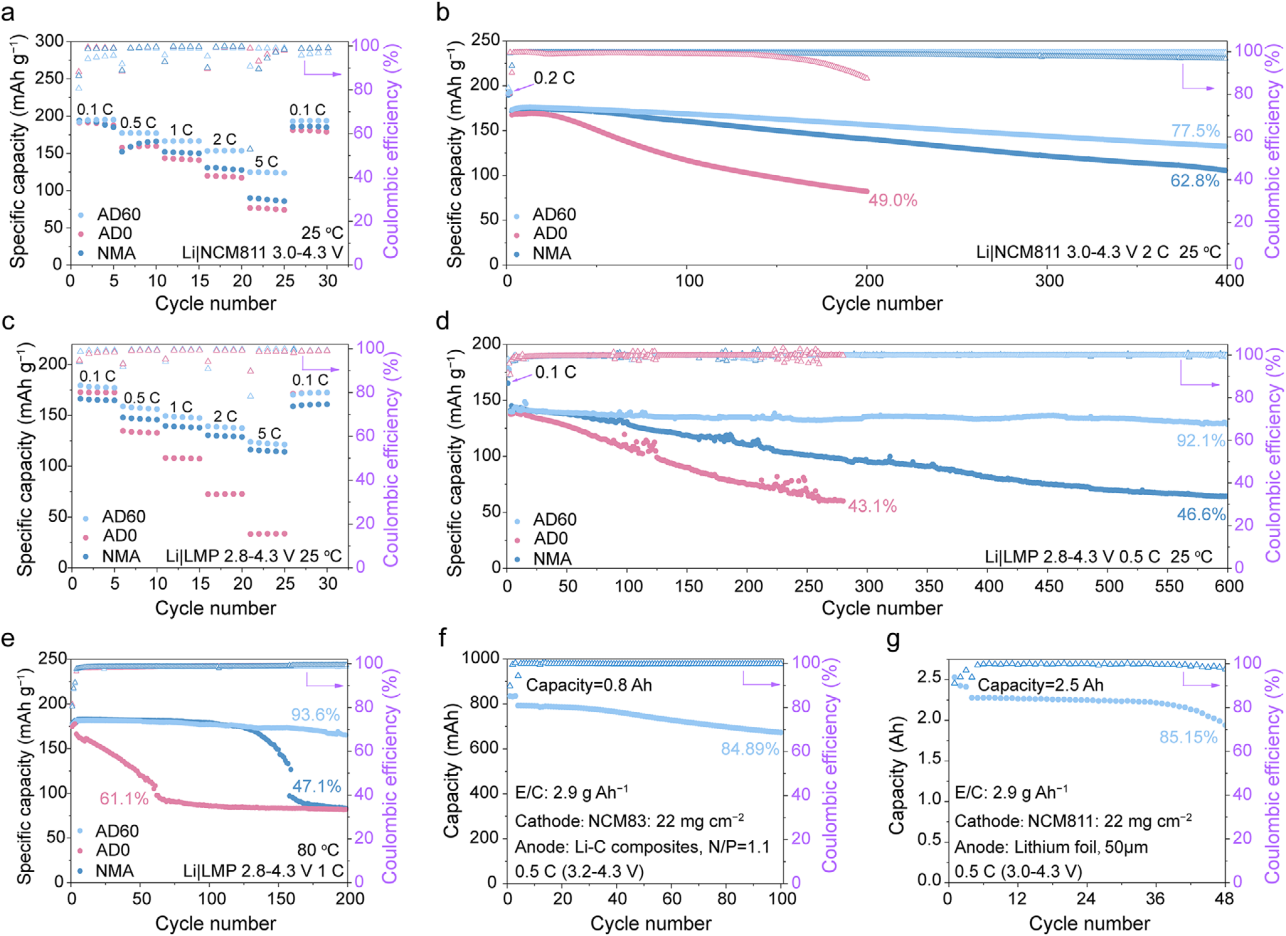
**Electrochemical performance of solid‐state lithium‐metal batteries using DEG electrolytes**. (a) Rate performance of the Li|NCM811 coin cells with three DEGs. (b) Long‐term cycling performance of Li|NCM811 coin cells at 2 C. (c) Rate performance of Li|LMP coin cells with three DEGs. (d) Long‐term cycling performance of Li|LMP coin cells at 0.5 C with three DEGs. (e) Long‐term cycling performance of Li|LMP coin cells at 1 C and 80°C (2.8–4.3 V). (f,g) Cycling performance and Coulombic efficiency of 0.8 Ah Li||Ni_0.83_Co_0.11_Mn_0.06_O_2_ (NCM83) and 2.5 Ah Li||NCM811 pouch cells using AD60 DEG, respectively.

To demonstrate the practical viability of our multifunctional electrolyte, we assembled and tested full cells using the optimized AD60 DEG with commercially relevant cathodes: LiMn_0.6_Fe_0.4_PO_4_ (LMP) and LiNi_0.8_Co_0.1_Mn_0.1_O_2_ (NCM811). The Li|AD60|NCM811 cell achieved a discharge capacity of 124.7 mAh g^−1^ at a high rate of 5 C (with 1 C defined as 200 mA g^−1^) (Figures 7a and S49). In contrast, the Li|NMA|NCM811 and Li|AD0|NCM811 cells retained only 90.02 and 76.63 mAh g^−1^ at 5 C, respectively. Long‐term cycling at 2 C (Figure 7b) confirms superior stability, with a high capacity retention of 77.5% (132 mAh g^−1^) over 400 cycles. The corresponding voltage profiles (Figure S50) show minimal polarization. Conversely, the control cells (AD0 and NMA) failed prematurely, with retentions of 49.0% and 62.8%, respectively (Figure S51).

The Li|AD60|LMP cell delivered a high reversible capacity of 123.8 mAh g^−^^1^ at a high rate of 5 C, significantly outperforming the NMA and AD0 control (Figure 7c). At 0.5 C, the Li|AD60|LMP cell retained 92.1% capacity after 600 cycles with an average coulombic efficiency of 99.67% (Figure 7d). Furthermore, as displayed in Figure S52, the Li|AD60|LMP cell exhibited stable voltage plateaus with minimal polarization throughout cycling, in contrast to the sharp capacity decline observed with the Li|NMA|LMP and Li|AD0|LMP cells. Given the excellent thermal stability and nonflammability of the DEG, we evaluated its performance at an elevated temperature of 80 °C. The Li|AD60|LMP cell maintained 93.6% of its initial capacity after 200 cycles at 1 C (Figure 7e). In contrast, the Li|NMA|LMP and Li|AD0|LMP cell systems exhibited significantly increased voltage hysteresis at 80 °C (Figure S53). This may be attributed to the fact that under high‐temperature conditions, the adverse reaction between active hydrogen and lithium metal is exacerbated, thereby impairing the interfacial stability of lithium metal. Similarly, unsatisfactory cycling performance of amide‐based electrolytes at high current rates has been reported in previous studies, which is attributed to the lack of protection from fluorinated groups.

We employed in situ EIS to monitor the evolution of impedance during delithiation and lithiation processes. The DRT spectra revealed three distinct peaks in both systems, each corresponding to a unique relaxation time and indicating a specific electrochemical process. Specifically, peaks observed at time constants of ∼10^−5^ s, ∼10^−4^ s, and 10^−3^–10^−2^ s were attributed to the charge transfer resistance (R*_ct_*), solid‐electrolyte interphase resistance (R*_SEI_*), and cathode‐electrolyte interface resistance (R*_CEI_*), respectively. The increasing impedance in the NMA‐based cell can be attributed to unstable R*_SEI_* and R*_CEI_* layers, whereas the fluorinated AD60 system maintained stable impedance, suggesting the formation of an inorganic‐rich, stable interphase that is crucial for long‐term cycling performance (Figures S54 and S55). To further evaluate the practical applicability of the AD60 DEG, 0.8 Ah Li||Ni_0.83_Co_0.11_Mn_0.06_O_2_ (NCM83) and 2.5 Ah Li||NCM811 pouch cells were assembled (Figures S56 and S57), with the detailed cell parameters summarized in Table S1. The 0.8 Ah pouch cell retained 84.89% of its capacity after 100 cycles, while the 2.5 Ah pouch cell retained 85.15% after 48 cycles, demonstrating the promise of the phase‐separated DEG electrolyte for lithium‐metal batteries (Figure 7f,g). The overall performance and practical cell‐level competitiveness of AD60 DEG are further benchmarked against representative gel polymer electrolytes in Tables S2 and S3. To further assess its tolerance to mechanical abuse and the associated risk of internal short circuits, a nail‐penetration test was conducted at a penetration speed of 25 mm s^−^^1^. As shown in Video S4, no flames or dense smoke were observed during the test. This behavior is consistent with the robust hydrogen‐bonding network and improved structural integrity of the gel electrolyte, which help suppress catastrophic failure under mechanical abuse.

## 3 Conclusion

In summary, we have developed a multifunctional deep eutectic gel electrolyte that successfully decouples the long‐standing trade‐off between mechanical robustness and ionic conductivity, delivering exceptional interfacial stability with lithium metal anodes. This was achieved through a facile one‐step strategy that utilizes molecular competition between polymer self‐association and polymer‐solvent solvation to drive the spontaneous formation of a bicontinuous and phase‐separated network. This unique architecture spatially integrates mechanical and transport functions, with rigid, PAM‐rich domains providing unprecedented strength and toughness, and soft, DES‐swollen PDMA‐rich channels facilitating rapid and selective Li^+^ transport. Furthermore, the incorporation of fluorinated TNMA facilitates the in situ formation of an inorganic LiF‐rich SEI, which promotes more uniform Li deposition and improves long‐term cycling stability compared with the nonfluorinated control system. The resulting electrolyte exhibits a synergistic combination of properties including high ionic conductivity (2.99 mS cm^−^^1^), an exceptional Li^+^ transference number (0.78), high tensile strength (11.4 MPa), excellent elasticity (473%), and outstanding stability in full cells. More broadly, this work establishes molecular competition as a powerful and generalizable design principle for programming the self‐assembly of advanced functional materials, offering a new paradigm for creating the next generation of multifunctional soft matter for applications ranging from energy storage to soft robotics and flexible electronics.

## Supporting Information

The authors have cited additional references within the Supporting Information [30, 31].

## Conflicts of Interest

The authors declare no conflicts of interest.

## Supporting information




**Supporting File 1**: adma74180‐sup‐0001‐SuppMat.docx.

**Supporting File 2**: adma74180‐sup‐0002‐MovieS1.mp4.

**Supporting File 3**: adma74180‐sup‐0003‐MovieS2.mp4.

**Supporting File 4**: adma74180‐sup‐0004‐MovieS3.mp4.

**Supporting File 5**: adma74180‐sup‐0005‐MovieS4.mp4.

## Data Availability

The data that support the findings of this study are available from the corresponding author upon reasonable request.
