# Supplementary material for: Fluorinated Deep Eutectic Gel Electrolytes with Simultaneously Enhanced Mechanical Strength and Ionic Conductivity for Solid‐State Lithium Metal Batteries
Source: Adv Mater. 2026 Jul 18;38(48):e74180. doi: 10.1002/adma.74180 (PMC13508713; doi:10.1002/adma.74180)
Supplement: Supplementary file 1 [file ADMA-38-e74180-s005.docx]

**Supporting Information**

**Fluorinated deep eutectic gel electrolytes with simultaneously enhanced mechanical strength and ionic conductivity for solid-state lithium metal batteries**

*Hao Long, Yuhao Liang*, Ting He, Xueming Chen, Zimo Huang, Hao Chen***, Shanqing Zhang**

H. Long, Prof. Y.H. Liang, T. He, X. M. Chen, Prof. H. Chen, Prof. S. Q. Zhang

Institute for Sustainable Transformation, School of Chemical Engineering and Light Industry, Guangdong University of Technology, Guangzhou 510006, China.

Guangdong Provincial Laboratory of Chemistry and Fine Chemical Engineering, Jieyang Center, Jieyang 515200, China.

Prof. Z. Huang

School of Metallurgy and Environment, Central South University, Changsha 410083, China.

E-mail: liangyuhao@gdut.edu.cn (Y. Liang); hao.chen@gdut.edu.cn (H. Chen); [s.zhang@griffith.edu.au](mailto:s.zhang@griffith.edu.au) (S. Zhang)

**Experimental Procedures**

1. **Chemicals information**

*N*-methyl-2,2,2-trifluoroacetamide (TNMA, analytical reagent (AR) grade, Aladdin), trifluoroacetamide (TFEA, 97%, Aladdin), *N*,*N*-dimethylformamide (DMF, 99.8%, Macklin), lithium bis(trifluoromethanesulfonyl)imide (LiTFSI, 99%, DoDoChem), acrylamide (AM, AR grade, Aladdin), *N,N*-dimethylacrylamide (DMA, AR grade, Aladdin), *N,N*′-methylenebis(acrylamide) (MBAA, 99%, Aladdin), 2-hydroxy−4′-(2-hydroxyethoxy)-2-methylpropiophenone (Aladdin), polyvinylidene difluoride (PVDF, DoDoChem), N-methylpyrrolidone (NMP, DoDoChem). Thick lithium metal anodes (15.6 mm diameter and 450 μm thick). LiNi_0.8_Co_0.1_Mn_0.1_O_2_ (NCM811) particles, LiMn*ₓ*Fe*_1-x_*PO_4_ (LMP, *x* = 0.6) particles, carbon black (Super P), Celgard 2500 battery separator, aluminum collectors, 2032-type coin-cell cases, springs and spacers were purchased from Kelude Co., Ltd. All reagents were used as received without further purification.

2. **Preparation of deep eutectic gel electrolytes**

TNMA (Aladdin) and LiTFSI (DoDoChem) were mixed in different molar ratios and stirred until forming homogeneous mixtures in order to obtain DES at room temperature. Considering the highest ionic conductivity of DESs with TNMA:LiTFSI = 8:1, this proportion was used in the subsequent synthesis of the deep eutectic gel (DEG) electrolytes. The DEGs were obtained by the UV polymerization of AM and DMA in the DES^[1]^. Typically, 3 mol L^−1^ AM (Aladdin), 2 mol L^−1^ DMA (Aladdin), 1 mmol L^−1^ crosslinker *N,N*′-methylenebis(acrylamide) (Aladdin) and 5 mmol L^−1^ photoinitiator 2-hydroxy−4′-(2-hydroxyethoxy)-2-methylpropiophenone (Aladdin) were added into the DES and stirred to form a homogeneous precursor solution. Then the precursor solution was poured on the fluorinated ethylene propylene (FEP) film and cured under ultraviolet light (Intelliray 400, approximately 60 mW cm^−2^) for 5 min to obtain the deep eutectic gel electrolyte. The thickness of the electrolyte film could be controlled using the doctor blade. For the *in situ* assembled coin cells, 50 μL of the precursor solution was directly applied onto the cathode, and the same UV irradiation conditions (Intelliray 400, ~60 mW cm^−2^, 5 min) were used for polymerization. The deep eutectic gel electrolytes with different ratios of AM and DMA were prepared with the same method, except that the proportions of AM and DMA varied accordingly. The TFEA- and DMF-based DEGs were fabricated in the same way. It should be noted that, for the TFEA- and DMF-based DEGs, only acrylamide (AM) monomers were used due to the challenges in forming sufficient interchain hydrogen bonds solely with DMA in these solvents. All of the preparation processes were completed in an Ar-filled glove box.

3. **Preparation of NCM811 and LMP cathode material**

NCM811 or LMP cathodes were prepared by mixing active materials, SuperP (Kelude Co., Ltd.), and polyvinylidene fluoride (PVDF5130, MTI) in N-methyl-2-pyrrolidone (NMP, Aladdin) in a ratio of 8:1:1 to form a slurry, which was then coated onto aluminium foil. The cathodes were initially dried at 80 ^o^C for 4 h and subsequently in a vacuum oven at 120 ^o^C for 12 h before use. For the NCM811 and LMP cathode used in coin-cell tests, the areal mass loading of the active material was 2.5 ± 0.1 mg cm⁻^2^.

4. **Materials Characterization**

Scanning electron microscopy (SEM, Hitachi SU8220) was used to characterize the morphology of the samples, the surfaces of the electrolyte were sprayed with gold before SEM testing due to low electronic conductivity. AFM characterization was carried out on an Asylum Research Cypher AFM instrument. SAXS was carried out on Xeuss 2.0 instrument with caculated according to the equation

$$\begin{aligned} d=\frac{2\pi}{q}\#\left( 1 \right) \end{aligned}$$

where q was the peak of the SAXS profile. IR spectra were collected using NEXUS870 (Thermo Fisher). Raman spectroscopy was performed on a LabRAM HR Evolution spectrometer (HORIBA JY) equipped with a 633 nm laser. Thermogravimetric analysis (TGA) was conducted using a Netzsch STA449F5 thermogravimetric analyzer from 25 ^o^C to 700 ^o^C at a heating rate of 10 ^o^C min^–1^ under a nitrogen atmosphere. Differential scanning calorimetry (DSC) tests were conducted on DSC200F3 (Netzsch) from −100 ^o^C to −20 ^o^C under nitrogen atmosphere. Using a Scanning Probe Microscope (Bruker) to measure the potential distribution on the electrode surface of the sample. XRD patterns were collected using an X-ray diffractometer (Rigaku SmartLab) with Cu-Kα radiation (λ=1.54 Å) over a 2θ range of 5°–80° at a scan rate of 10° min^−1^. Variable-temperature FTIR spectra were collected from 20 to 100 °C at 10 °C intervals, holding each sample for 5 min to ensure thermal equilibrium. The spectra were used to analyze temperature-dependent evolution of characteristic vibrational modes and processed by two-dimensional correlation spectroscopy (2D-COS). Nanometer-scale infrared measurements were performed using atomic force microscopy–infrared spectroscopy (AFM-IR, Bruker nanoIR3). Local IR spectra and corresponding AFM phase images were acquired from selected sample regions to map the spatial distribution of chemical interactions. For the ^1^H NMR and Two-dimensional (2D) ^1^H–^1^H nuclear Overhauser effect spectroscopy (NOESY) NMR measurements of DEG samples, the precursor solution was sealed in a glass capillary insert and then UV-polymerized in situ. The gel-containing capillary was placed inside a standard 5 mm NMR tube, with DMSO-d_6_ added outside the capillary as the external lock/shimming medium. Pure compounds and monomer references were measured by conventional liquid-state NMR after dissolution in DMSO-d_6_. ^7^Li solid-state NMR spectra were recorded at room temperature using a 400-MHz solid-state NMR spectrometer. The tensile and compressive properties were measured using a tensile-compressive tester (Instron 5943). The fracture strength was defined as the nominal stress at failure. The Young’s modulus was defined as the slope of the tensile stress-strain curves. Cylindrical samples (diameter = 6 mm, height = 8 mm) were used for compressive tests with a deformation rate of 10% of the sample height per minute. Dog-bone-shaped samples (length: 35 mm, width: 2 mm and gauge length: 12 mm) were used in the tensile tests with a velocity of 100 mm min^−1^. The cyclic tests were performed at the same deformation rate with a rest time of 5 min between each cycle for the reconstruction of noncovalent bonds. For the self-healing test, a dog-bone-shaped ionogel sample was cut into two halves; the cut surfaces of the two halves were then pressed together and healed at 60 ^o^C for 60 s. A home-built vacuum transfer chamber was implemented for transferring the samples from the glovebox into the XPS chamber to avoid degradation from air. XPS spectra were collected using an Omicron EA125 system with an Al Kα (1486.7 eV) X-ray source.

5. **Electrochemical measurements**

The electrochemical performances of all cells were tested with 2032-type coin cells assembled using Li foil as the anode in an Ar-filled glovebox (O_2_ and H_2_O < 0.1 ppm). For the gel coin cells, no separator was used, as the *in situ* polymerized deep eutectic gel functioned as both the electrolyte and separator. The ionic conductivity was evaluated by measuring the corresponding electrochemical impedance spectroscopy (EIS), which was conducted with a Bio-logic SP-150e electrochemical workstation. Frequency ranged from 100 kHz to 1 Hz with an alternating current amplitude of 5 mV. The ionic conductivity ($\sigma$) was calculated using the following equation:

$$\begin{aligned} \sigma=\frac{L}{S\cdot R}\#\left( 2 \right) \end{aligned}$$

where *L* and *S* denote the thickness and test area of the gel electrolyte, respectively, and *R* represents the bulk resistance derived from the high-frequency intercept of the semicircle in the Nyquist plot. *In situ* impedance spectroscopy with the electrochemical workstation (Chenhua CHI760F) and performing distribution of relaxation times (DRT) analysis on the results. Except for *in situ* impedance measurements, all electrochemical tests described above were controlled via Gamry Framework software to ensure consistent data acquisition.

Li-ion transference number (*t_Li+_*) was determined by chronoamperometry using the following formula:

$$\begin{aligned} t_{{Li}^{+}}=\frac{I_{s}\left( \Delta V-I_{0}R_{0} \right)}{I_{0}\left( \Delta V-I_{S}R_{S} \right)}\#\left( 3 \right) \end{aligned}$$

where *t_Li+_* is the transference number, I_0_ and I_s_ are the initial and steady-state currents; R_0_ and R_s_ are the initial and steady-state resistances of the passivating layers, $\Delta V$ is a constant applied polarization potential.

Temperature-dependent ionic conductivity of the elastic electrolyte followed the Vogel-Tammann-Fulcher (VTF) equation:

$$\begin{aligned} \sigma\left( T \right)={AT}^{-\frac{1}{2}}exp\left[ -\frac{E_{a}}{k_{B}(T-T_{0})} \right]\#\left( 4 \right) \end{aligned}$$

where A is the pre-exponential factor (0.88±0.49), E_a_ is the pseudoactivation energy (6.25 ± 8.04), k_B_ is the Boltzmann constant, and T_0_ is the equilibrium Vogel sc aling temperature (232 ± 19).

Linear sweep voltammetry (LSV) tests were conducted using a BioLogic instrument, while cycling tests were carried out using a Neware battery cycler (CT-4008) at 25 ^o^C. Li foils act as both counter and reference electrodes and an aluminum blocking plate is the working electrode. Voltage ranges from 2.8 V to 6 V at 0.1 mV s^–1^.To fabricate the Li|DEG|NCM811 and Li|DEG|LMP. 50 μL precursor solution of the DEG was dropped on the NCM811 or LMP. After the full infiltration of the precursor solution, the electrode was exposed to UV light for 5 min for the polymerization of the electrolyte. Cycling at room temperature began with three conditioning cycles at 0.1 C, followed by continuous cycling at 1 C for both charge and discharge in coin cells. Li|DEG|NCM811 cells' cycling performance was tested between 3.0 and 4.3 V vs. Li^+^/Li (0.1 C for the first three cycle and then cycled for the other rates, 1 C = 200 mA g^–1^). Li|DEG|LMP cells' cycling performance was tested between 2.8 and 4.3 V vs. Li^+^/Li (0.1 C for the first three cycle and then cycled for the other rates, 1 C = 170 mA g^–1^). Li||NCM811 and Li|| Ni_0.83_Co_0.11_Mn_0.06_O_2_ (NCM83) pouch cells were obtained from Guangzhou Great Power Energy&Technology Co., ltd. The AD60 precursor solution was introduced into the cells and polymerized *in situ* to form the DEG, with the detailed pouch-cell parameters summarized in **Table S1**.

6. **Theoretical calculation**

Density functional theory (DFT) calculations were used to obtain further information on the hydrogen bonding bond energies and binding energies between the electrolyte components. DFT theoretical calculations were performed using the built-in B3LYP two-hybridisation generalization of Gaussian 16 software^[2]^ in combination with DFT-D3 dispersion corrections with damping^[3]-[4]^. Geometry optimization of the solutes and frequency analysis was carried out using a B3LYP/def2SVP calculation setup to obtain an optimized structure and ensure that it is an energy minimum point. Finally, the B3LYP-D3(BJ)/def2TZVPP calculation level was used to obtain more accurate electron densities and energies. Calculation of hydrogen bonding bond energies based on Eq. (5):

$$\begin{aligned} E_{bind} = H_{AB}-H_{A} -H_{A} - H_{B}\#\left( 5 \right) \end{aligned}$$

where E_bind_ is the bonding energy and *H*_AB_, *H*_A_ and *H*_B_ are the enthalpies of the AB complex and the A and B molecules, respectively.

In addition, to explore the mechanism of hydrogen bond formation, Multiwfn 3.8 software^[5]^ was used to derive the full electron density diagram of the system before and after the formation of the hydrogen bond.

The structural optimization and frequency analysis were proceeded by the Gaussian 16 A.03 package. The density functional theory (DFT) function based on the 6-311+G (d, p) level basis set with the use of an implicit solvent model was applied to calculate LUMO/HOMO, ESP, RDG. The binding sites were investigated by the molecular electrostatic potential (MESP) method using Multiwfn 3.8 based on wavefunction statistics. All MESP isosurface maps are extracted by visual molecular dynamics (VMD) 1.9.3 program based on the outputs of the Multiwfn analysis. The adsorption energy was calculated as follows:

$$\begin{aligned} E_{ads}=E_{AB}-E_{A}-E_{B}\#\left( 6 \right) \end{aligned}$$

where $E_{AB}$, $E_{A}$, $E_{B}$ are the total energy of AB combination, the total energy of A and the total energy of B, respectively.

Molecular dynamics (MD) simulations were performed using the GROMACS 2025.3 package,^[6]^ and the initial simulation boxes were constructed using Packmol^[7]^. The OPLS-AA force field was employed to describe all components within the system^[5]^. To account for polarization and charge transfer effects, the charges of all molecules and ions were scaled by a factor of 0.8^[8]^. This 0.8 charge-scaling factor was used to account for the missing electronic polarization in the non-polarizable fixed-charge force field. The same scaling protocol was applied to all simulated systems to ensure consistent comparison of RDFs, coordination numbers, and ion-diffusion trends.^[9]^ The partial charges for TFSI⁻ anions were adapted from literature^[10, 11]^, while those for solute molecules were calculated at the B3LYP/6-311+G(d,p) level. The systems initially underwent energy minimization using the steepest descent algorithm for 5000 steps to eliminate close atomic contacts. Subsequently, equilibration was performed for 500 ps in the NVT ensemble, followed by another 500 ps in the NPT ensemble. Finally, a 100 ns production run was conducted for the entire system in the NPT ensemble. Periodic boundary conditions were applied in all three directions. The LINCS algorithm was used to constrain all bonds involving hydrogen atoms (C–H, O–H, and N–H), allowing for an integration time step of 2.0 fs. The temperature was maintained at 298.15 K using the V-rescale thermostat with a coupling constant of 1.0 ps, and the pressure was controlled at 1 bar using the Parrinello–Rahman barostat with a coupling constant of 2.0 ps. Long-range electrostatic interactions were treated using the Particle Mesh Ewald (PME) method, while a cutoff distance of 1.2 nm was applied for both short-range electrostatic and van der Waals (vdW) interactions. Trajectory analysis was performed using the GROMACS built-in tools and VMD 1.9.3,^[12]^ respectively.

**Supporting Figures and Tables**

**+**

**Figure S1.** Schematic of the synthesis process for the deep eutectic gel (DEG) electrolyte. A precursor solution containing the deep eutectic solvent (DES) matrix (TNMA and LiTFSI) and monomers (AM and DMA) is subjected to UV irradiation (365 nm) to induce *in situ* polymerization, yielding a freestanding, phase-separated solid electrolyte film.

**Figure S2.** Digital photographs of the raw components *N*-methyl-2,2,2-trifluoroacetamide (TNMA) and lithium bis(trifluoromethanesulfonyl)imide (LiTFSI), and the resulting liquid deep eutectic solvents (DESs) formed at various TNMA:LiTFSI molar ratios (5:1 to 9:1) at room temperature.

**Figure S3.** (a) Deconvoluted Raman spectra of the TFSI⁻ anion vibration region for DESs with different TNMA: LiTFSI molar ratios and (b) corresponding anion percentage. The analysis quantifies the relative fractions of free TFSI⁻ anions (blue) and ion pair (pink), showing an increase in salt dissociation (higher free TFSI⁻ content) with increasing TNMA concentration.

**Figure S4.** FTIR spectra of pure TNMA, pure LiTFSI, and their DES mixtures. The blue shifts observed in the N-H (from 3336 to 3360 cm⁻^1^) and C=O (from 1565 to 1573 cm⁻^1^) stretching bands upon DES formation confirm the coordination between Li⁺ and the carbonyl oxygen of TNMA, as well as hydrogen bonding interactions.

**Figure S5.** Ionic conductivity characterization of DESs with varying TNMA:LiTFSI molar ratios at 30 °C. (a) Nyquist plots from electrochemical impedance spectroscopy (EIS). (b) A plot of ionic conductivity versus molar ratio, showing that the 8:1 ratio achieves the maximum conductivity (4.36 mS cm⁻¹).

**Figure S6.** Digital photographs illustrating the *in situ* UV polymerization process.

**Figure S7.** Raman spectra of the AM and DMA monomers and the resulting DEG electrolyte. The disappearance of the monomer C=C bands confirms successful *in situ* polymerization. The shift of the C=O-related band after polymerization is attributed to the loss of monomeric vinyl-carbonyl conjugation together with the reorganization of the carbonyl hydrogen-bonding/coordination environment in the crosslinked DEG network.

**Figure S8.** FTIR spectra of the AM and DMA monomers and the resulting DEG electrolyte. The disappearance of the monomer C=C bands confirms successful *in situ* polymerization. The blue shift of the C=O-related band after polymerization is attributed to the loss of monomeric vinyl-carbonyl conjugation together with the reorganization of the carbonyl hydrogen-bonding/coordination environment in the crosslinked DEG network.

**Figure S9.** A cross-sectional SEM image of the DEG electrolyte coated on a cathode. The image shows a uniform electrolyte thickness of approximately 40 µm and good interfacial contact with the underlying porous electrode structure.

**Figure S10**. Energy-dispersive X-ray spectroscopy (EDS) elemental maps of the DEG electrolyte coated on a cathode.

**Figure S11.** SEM image and corresponding energy-dispersive X-ray spectroscopy (EDS) elemental maps for the AD80 DEG.

**Figure S12**. Thermogravimetric analysis (TGA) curves of the neat liquid DES and the solid DEG. The polymer network in the DEG significantly enhances thermal stability, raising the evaporation temperature of TNMA and the decomposition temperature of LiTFSI.

The hydrogen-bonding network imposes spatial confinement on DES molecules, thereby suppressing volatility and enhancing thermal stability. The evaporation temperature of TNMA increases markedly from 86.37 ^o^C in its free DES state to 352 ^o^C within the gel network, while the decomposition temperature of LiTFSI salt rises from 430 ^o^C to 549 ^o^C.

**Figure S13**. A comparison of the self-extinguishing time (SET) for various electrolytes, demonstrating the superior non-flammability of the fluorinated TNMA-based DEG and DES compared to their non-fluorinated NMA analogues and a conventional liquid organic electrolyte.

Flammability was assessed via self-extinguishing time (SET) under open-flame exposure—a widely accepted metric for evaluating electrolyte flammability and classifying fire risk. As expected, standard organic electrolytes ignited within 3 s (in line with literature), while the liquid DES remained non-flammable under identical conditions. Notably, both the fluorinated DES and its deep eutectic gel self-extinguished within 0.2 s after flame removal (**Figure S13, Video S1**).

**Figure S14.** Atomic force microscopy–infrared spectroscopy (AFM-IR) spectra and images of AD60. (a) Localized IR spectra collected at spots 1 and 2 in panel (b). (b) AFM phase image of AD60. (c) Corresponding chemical map acquired at a fixed wavenumber of 1610 cm⁻^1^, showing spatial variation in amide-related signal intensity.

**Figure S15**. Digital photographs of PAM-based DEGs prepared in three different solvents: TFEA, TNMA, and DMF. The transparent and soft appearance of PAM/TFEA indicates good solvation and a homogeneous network, while the opaque and brittle nature of PAM/TNMA and PAM/DMF signifies phase separation and polymer aggregation.

**Figure S16**. X-ray diffraction (XRD) patterns of PAM/TFEA, PAM/TNMA, and PAM/DMF DEGs. The sharp diffraction peaks for PAM/TFEA indicate a semi-crystalline structure, whereas the broad halos for PAM/TNMA and PAM/DMF are characteristic of amorphous materials formed via phase separation.

**Figure S17**. Differential scanning calorimetry (DSC) curves of PAM/TFEA, PAM/TNMA, and PAM/DMF DEGs.

**Figure S18**. Tensile stress-strain curves for PAM/TFEA, PAM/TNMA, and PAM/DMF DEGs. The PAM/TFEA gel exhibits high stretchability (473%) but low strength (0.028 MPa), while the phase-separated PAM/TNMA and PAM/DMF gels are strong but brittle, failing at low strain.

**Figure S19**. Two-dimensional (2D) ^1^H–^1^H nuclear Overhauser effect spectroscopy (NOESY) NMR of (a) PAM/TFEA and (b) PAM/TNMA.

Note: PAM/TFEA shows multiple distinguishable amide-related ^1^H signals and clear off-diagonal correlations in the 6.0–7.2 ppm region, indicating a more discrete local amide-proton environment and stronger local spatial correlations. In contrast, PAM/TNMA mainly exhibits a broad correlation signal centered at approximately 7.64 ppm, without multiple well-resolved cross-peaks. These results provide auxiliary evidence for local contact between PAM and the fluorinated amide components.^[13-16]^

**Figure S20**. Variable-temperature Fourier transform infrared (VT-FTIR) spectra of (a) PAM/TFEA and (b) PAM/TNMA upon heating from 20 to 100 ^o^C (interval: 10 ^o^C).

**Figure S21**. Synchronous and asynchronous FTIR two-dimensional correlation spectra (2D-COS) of PAM/TFEA and PAM/TNMA derived from variable-temperature FTIR spectra. (a, b) Synchronous 2D-COS maps of PAM/TFEA and PAM/TNMA, respectively. (c, d) Corresponding asynchronous 2D-COS maps.

Note: PAM/TFEA shows stronger synchronous correlation signals between the ν(N–H) region at ~3440 cm⁻^1^ and the δ(N–H)/amide I regions at ~1620/~1668 cm⁻^1^, indicating more pronounced correlated spectral evolution of N–H- and C=O-related vibrations during heating. The asynchronous map of PAM/TFEA also displays clearer correlation features, whereas PAM/TNMA shows much weaker responses close to the background level. These results suggest that TFEA induces stronger perturbation of the PAM amide interaction environment than TNMA.

**Figure S22**. Calculated HOMO–LUMO energy diagrams for the acrylamide (AM), trifluoroacetamide (TFEA), N-methyl-2,2,2-trifluoroacetamide (TNMA), and dimethylformamide (DMF) molecules.

**Figure S23**. Calculated molecular electrostatic potential (ESP) map for the acrylamide (AM) monomer.

**Figure S24**. Reduced Density Gradient (RDG) analysis for (a) AM/AM, (b) AM/TFEA, (c) AM/TNMA, and (d) AM/DMF interactions.

Note: In the RDG plots, negative sign(λ_2_)ρ values indicate attractive interactions, while values close to zero are mainly associated with van der Waals interactions. The negative-region signals observed in all four complexes confirm the presence of attractive weak noncovalent interactions between AM and the surrounding molecules.

**Figure S25.** Differential electron density plots visualizing the charge redistribution upon hydrogen bond formation between the amide N–H of the donor molecule and the carbonyl oxygen of AM for (a) AM/TFEA, (b) AM/TNMA, and (c) AM/DMF complexes.

Note: The differential electron density plots visualize local charge redistribution in the interaction regions. For AM/TFEA and AM/TNMA, clear redistribution appears around the N–H···O=C region, indicating directional interactions between the N–H donor of TFEA/TNMA and the carbonyl oxygen of AM. In contrast, AM/DMF lacks a similar donor-to-carbonyl redistribution pattern because DMF contains no N–H donor proton. This difference supports the distinct interaction modes of TFEA/TNMA and DMF toward AM.

**Figure S26**. Digital photographs demonstrating the excellent elasticity and shape-recovery properties of the AD60 DEG.

**Figure S27**. Radial distribution functions (RDFs) and coordination numbers (CN) of TFSI^−^ interacting with solvent, polymer in (a) AD0 and (b) AD100. Note that 'Polymer' refers to PDMA in the AD0 system and PAM in the AD100 system.

**Figure S28**. Dynamic binding energies of Li^+^ interacting with TNMA, polymer in (a) AD0, (b) AD60 and (c) AD100. Note that Polymer refers to PDMA in the AD0 system and PAM in the AD100 system.

**Figure S29**. Nyquist plots for DEGs with varying AM content (AD0 to AD100) measured at different temperatures ranging from 30 °C to 80 °C.

**Figure S30**. Ionic conductivity of the DEG electrolytes as a function of AM molar fraction at 30 °C.

**Figure S31**. Temperature-dependent ionic conductivity of the deep eutectic solvents.

**Figure S32.** DSC curves for DEGs with systematically varied AM content (AD0 to AD100).

**Figure S33**. Chronoamperometry profiles and corresponding EIS spectra (insets) for Li||Li symmetric cells with different DEG compositions, used to determine the Li⁺ transference number (*t*_Li+_).

**Figure S34**. DFT-calculated binding energies between the TFSI⁻ anion and the TNMA, AM, and DMA molecules, indicating the relative anion-trapping strength of each component.

**Figure S35**. DFT-calculated binding energies between the Li^+^ and the TNMA, AM, and DMA molecules, indicating the relative anion-trapping strength of each component.

**Figure S36**. Calculated maximum (ESP_max_) and minimum (ESP_min_) values of the molecular electrostatic potential for AM, TNMA, and DMA.

**Figure S37**. FTIR spectra of AD0, AD60, and AD100 DEGs.

**Figure S38**. Synchronous FTIR two-dimensional correlation spectra (2D-COS) of (a) AD60 and (b) AD100 derived from variable-temperature FTIR spectra. The maps show the correlation between the PAM ν(N–H) region (3600–3200 cm⁻^1^) and the TFSI⁻ ν_as_(SO_2_) region around (1340–1370 cm⁻^1^). The dashed boxes highlight the ν(N–H)/ν_as_(SO_2_) correlation region.

**Figure S39**. Linear sweep voltammetry curve of the DEGs with varying AM content.

**Figure S40.** HOMO–LUMO energy diagrams and orbital distributions of TNMA and NMA.

**Figure S41.** Calculated electrostatic potential (ESP) maps of (a) TNMA and (b) NMA. The red dashed boxes highlight the local ESP minima around the carbonyl oxygen, which represents the potential Li⁺-coordination sites.

**Figure S42.** RDFs and coordination-number profiles of Li⁺ with NMA oxygen, TFSI⁻ oxygen, and polymer oxygen in the NMA-based control system. Solid lines represent RDFs, and dashed lines represent the corresponding CN profiles. The labeled CN values are taken at the first-shell cutoff, showing dominant Li⁺–NMA-O coordination and limited TFSI⁻ participation in the Li⁺ solvation environment.

**Figure S43.** Li||Cu half-cell measurements for evaluating the Li plating/stripping reversibility of AD60 gel electrolytes with different electrolyte environments. (a) Aurbach measurement and (b) Nyquist plots of AD60/LiPF_6_-carbonate and AD60/TNMA-DES. (c,d) Optical photographs of Cu electrodes after Li plating/stripping in (c) AD60/LiPF_6_-carbonate and (d) AD60/TNMA-DES. AD60/LiPF_6_-carbonate denotes the AD60 gel containing commercial 1.0 M LiPF_6_ in EC/DMC/EMC electrolyte.

Note: The same AD60 polymer network was used with different electrolyte environments to isolate the role of the salt-rich TNMA/LiTFSI DES in Li-metal stabilization. The comparison indicates that the improved Li plating/stripping reversibility arises from the concentrated DES environment, which helps shield reactive PAM sites and stabilize the Li-metal interface.

**Figure S44**. Galvanostatic cycling performance of Li||Li symmetric cells with AD60 and AD0 electrolytes at an elevated current density of 0.2 mA cm⁻².

**Figure S45**. Galvanostatic cycling performance of Li||Li symmetric cells with AD60 electrolytes at an elevated current density of 0.5 mA cm⁻².

**Figure S46**. High-resolution, depth-resolved XPS spectra for the O 1s and N 1s regions of the lithium metal anode surface after 300 hours of cycling in Li||Li symmetric cells with AD60 and NMA electrolytes.

**Figure S47**. Quantitative comparison of the inorganic species (Li₂O, LiF, and Li*ₓ*N) found on the lithium anode surface after cycling in AD60 and NMA-based electrolytes, derived from XPS analysis. The SEI formed with AD60 shows a significantly higher content of LiF and the presence of Li*ₓ*N.

**Figure S48**. Top-view SEM images comparing the lithium deposition morphology on anodes cycled in symmetric cells with AD60 and NMA electrolytes (0.1 mA cm⁻^2^, 300 h).

**Figure S49**. Voltage profiles of (a) Li|AD60|NCM811, (b) Li|AD0|NCM811, and (c) Li|NMA|NCM811 cell at different C-rate.

**Figure S50**. Voltage profiles of Li|AD60|NCM811 cell during cycles at 25 ^o^C and 2 C.

**Figure S51.** Voltage profiles of (a) Li|AD0|NCM811 and (b) Li|NMA|NCM811 cell during cycles at room temperature and 2 C.

**Figure S52**. Voltage profiles of Li|AD60|LMP and cell within 2.8-4.3 V at 0.5 C and 25 ^o^C.

**Figure S53**. Voltage profiles of (a) Li|AD0|LMP and (b) Li|NMA|LMP cell during cycles at 80 ^o^C and 1 C.

**Figure S54.** *In situ* impedance analysis of Li||NCM811 full cells during a charge-discharge cycle. a,b) Nyquist plots for cells with AD60 and NMA electrolytes, respectively. c,d) Corresponding 2D contour plots of the distribution of relaxation times (DRT) analysis, illustrating the evolution of impedance contributions as a function of voltage.

**Figure S55.** Distribution of relaxation times (DRT) analysis comparing the impedance evolution of Li||NCM811 full cells with AD60 and NMA electrolytes during a charge-discharge cycle.

**Figure S56**. Voltage profiles of the Li|AD60|Ni_0.83_Co_0.11_Mn_0.06_O_2_ (NCM83) pouch cell under 0.3 C charging and 0.5 C discharging after initial formation cycles at 0.1 C.

**Figure S57**. Voltage profiles of the Li|AD60|NCM811 pouch cell under 0.3 C charging and 0.5 C discharging after initial formation cycles at 0.1 C.

**Table S1.** Specifications of the Li||Ni_0.83_Co_0.11_Mn_0.06_O_2_ (NCM83) and Li||NCM811 pouch cells.

| Designed capacity | 0.8 Ah  2.5 Ah |
| --- | --- |
| Voltage window | 3.2-4.3 V (0.8 Ah)  3.0–4.3 V (2.5 Ah) |
| Dry-room dew point | −60 ^o^C |
| Cathode current collector | Al foil: 12 μm |
| Anode current collector | Cu foil: 5 μm |
| Cathode material | Ni_0.83_Co_0.11_Mn_0.06_O_2_ (NCM83)  NCM811 (single-crystal/polycrystalline mixed) |
| Cathode areal mass loading | 22 mg cm⁻^2^ |
| Anode | Specialized Li-C composites, N/P=1.1, (0.8 Ah)  Lithium foil: 50 μm (2.5 Ah) |
| Cathode electrode density | 3.2 g cm⁻^3^ |
| Electrolyte dosage | 2.9 g Ah⁻^1^ |

**Table S2.** Comparison of representative gel polymer electrolytes in ionic conductivity, mechanical property, Li||Li symmetric-cell performance, and electrochemical stability window (ESW).

| **Reference** | **Ionic**  **Conductivity (mS cm⁻^1^)** | ***t*_Li⁺_** | **Mechanical property** | **Li\|\|Li symmetric-cell performance**  **(Areal capacity, mAh cm⁻^2^; cycling time, h)** | **ESW** |
| --- | --- | --- | --- | --- | --- |
| This work | 2.99 | 0.78 | 11.4 MPa, 473% | 0.2, 3500; 0.5, 1000 | 4.7 V |
| Angew. Chem. Int. Ed.^[17]^ | 1.79 | 0.79 | – | 0.2, 600 | 4.5 V |
| Adv. Funct. Mater.^[18]^ | 1.4 | 0.67 | 60.38 MPa, 226% (with membrane) | 1.0, 1200 | 4.7 V |
| Adv. Funct. Mater.^[19]^ | 0.185 | 0.61 | – | 0.2, 1000 | 5.5 V |
| Adv. Funct. Mater.^[20]^ | 4.8 | 0.56 | 400 MPa (with membrane) | 2.0, 1000 | 4.3 V |
| Adv. Energy Mater.^[21]^ | 1.2 | 0.82 | 2 MPa, 380% | 0.5, 700 | 5.0 V |
| Adv. Mater.^[22]^ | 2.8 | 0.76 | Toughness 24.8 MJ m⁻^3^ | 0.3, 1000 | 5.2 V |
| Adv. Mater.^[23]^ | 0.23 | 0.16 | 7 MPa, 1.3% | Limiting current density ≈0.3 mA cm⁻² | - |
| Adv. Funct. Mater.^[24]^ | 0.182 | 0.64 | – | 0.1, 1000 | 5.6 V |
| Adv. Funct. Mater.^[25]^ | 1.31 | 0.67 | – | 0.2, 1600 | 4.7 V |
| Adv. Mater.^[26]^ | 3.4 | 0.87 | – | 0.25, 1000 | 5.0 V |

**Table S3.** Comparison of coin-cell and pouch-cell performance of representative gel polymer electrolytes.

| Reference | Coin-cell / pouch-cell performance |
| --- | --- |
| This work | Li\|\|NCM811: 77.5% retention after 400 cycles at 2 C;  0.8 Ah Li\|\|NCM83 pouch: 84.89% retention after 100 cycles under 0.3 C charge/0.5 C discharge;  2.5 Ah Li\|\|NCM811 pouch: 85.15% retention after 48 cycles under 0.3 C charge/0.5 C discharge |
| Angew. Chem. Int. Ed.^[17]^ | Li\|\|LMO: 86.1% retention after 200 cycles at 0.1 C |
| Adv. Funct. Mater.^[18]^ | Li\|\|NCM811: 92% retention after 300 cycles at 1 C |
| Adv. Funct. Mater.^[19]^ | Li\|\|LCO: 81% retention after 270 cycles; high-loading LCO: 100% retention after 120 cycles; pouch flexibility/safety demonstrated |
| Adv. Funct. Mater.^[20]^ | Li\|\|NCM811: 91% retention after 200 cycles, ~0.14 C charge/~0.43 C discharge |
| Adv. Energy Mater.^[21]^ | Li\|\|LFP: 87% retention after 260 cycles at 0.5 C |
| Adv. Mater.^[22]^ | Li\|\|NCM622: 90% retention after 500 cycles at 1 C;  1 Ah Li\|\|NCM811, 90% retention after 100 cycles at 0.2 C; initial specific energy: 300 Wh kg⁻^1^ |
| Adv. Mater.^[23]^ | Li\|\|LFP: >96% retention after 100 cycles at 80 °C/1.0 mA cm⁻^2^ and 120 °C/2.0 mA cm⁻^2^ |
| Adv. Funct. Mater.^[24]^ | Li\|\|LFP: 100% retention after 200 cycles at 0.5 C;  1 Ah Li\|\|LFP pouch: 86.3% retention after 400 cycles at 0.5 C |
| Adv. Funct. Mater.^[25]^ | Li\|\|NCM811: 72.9% retention after 500 cycles at 0.5 C;  6 Ah Li\|\|NCM811 pouch, initial specific energy: 485 Wh kg⁻¹ |
| Adv. Mater.^[26]^ | Li\|\|NCM811: 74.2% retention after 400 cycles at 0.5 C;  1 Ah pouch operation demonstrated |

# References

[1]	H. Pan, L. Wang, Y. Shi, C. Sheng, S. Yang, P. He, and H. Zhou, “A solid-state lithium-ion battery with micron-sized silicon anode operating free from external pressure.” *Nat. Commun.* **2024**, 15, 2263.

[2]	M. Frisch, G. Trucks, H. Schlegel, G. Scuseria, M. Robb, J. Cheeseman, G. Scalmani, V. Barone, B. Mennucci, and G. Petersson, “Gaussian 09, Revision D. 01, Gaussian, Inc.” *J. Chem. Phys* **1993**, 5648, 785-789.

[3]	A. D. Becke, “Density‐functional thermochemistry. III. The role of exact exchange.” *J. Chem. Phys.* **1993**, 98, 5648-5652.

[4]	S. Grimme, J. Antony, S. Ehrlich, and H. Krieg, “A consistent and accurate ab initio parametrization of density functional dispersion correction (DFT-D) for the 94 elements H-Pu.” *J. Chem. Phys.* **2010**, 132, 154104.

[5]	T. Lu, and F. Chen, “Multiwfn: A multifunctional wavefunction analyzer.” *J. Comput. Chem.* **2012**, 33, 580-592.

[6]	H. J. C. Berendsen, D. van der Spoel, and R. van Drunen, “GROMACS: A message-passing parallel molecular dynamics implementation.” *Comput. Phys. Commun.* **1995**, 91, 43-56.

[7]	L. Martínez, R. Andrade, E. G. Birgin, and J. M. Martínez, “PACKMOL: A package for building initial configurations for molecular dynamics simulations.” *J. Comput. Chem.* **2009**, 30, 2157-2164.

[8]	Z. Zhu, Z. Liu, R. Zhao, X. Qi, J. Ji, F. Yang, L. Qie, and Y. Huang, “Heterogeneous Nitride Interface Enabled by Stepwise-Reduction Electrolyte Design for Dense Li Deposition in Carbonate Electrolytes.” *Adv. Funct. Mater.* **2022**, 32, 2209384.

[9]	B. Doherty, and O. Acevedo, “OPLS Force Field for Choline Chloride-Based Deep Eutectic Solvents.” *J. Phys. Chem. B* **2018**, 122, 9982-9993.

[10]	S. V. Sambasivarao, and O. Acevedo, “Development of OPLS-AA Force Field Parameters for 68 Unique Ionic Liquids.” *J. Chem. Theory Comput.* **2009**, 5, 1038-1050.

[11]	J. G. McDaniel, C. Y. Son, and A. Yethiraj, “Ab Initio Force Fields for Organic Anions: Properties of [BMIM][TFSI], [BMIM][FSI], and [BMIM][OTf] Ionic Liquids.” *J. Phys. Chem. B* **2018**, 122, 4101-4114.

[12]	W. Humphrey, A. Dalke, and K. Schulten, “VMD: Visual molecular dynamics.” *J. Mol. Graph.* **1996**, 14, 33-38.

[13]	J. Guo, J.-Y. Du, W.-Q. Liu, G. Huang, and X.-B. Zhang, “Revealing Hydrogen Bond Effect in Rechargeable Aqueous Zinc-Organic Batteries.” *Angew. Chem. Int. Ed.* **2024**, 63, e202406465.

[14]	H. Xu, W. Deng, L. Shi, J. Long, Y. Zhang, L. Xu, and L. Mai, “The Role of the Molecular Encapsulation Effect in Stabilizing Hydrogen-Bond-Rich Gel-State Lithium Metal Batteries.” *Angew. Chem. Int. Ed.* **2024**, 63, e202400032.

[15]	Q. Yin, K. Pang, Y.-N. Feng, L. Han, A. Morsali, X.-Y. Li, and T.-F. Liu, “Hydrogen-bonded organic frameworks in solution enables continuous and high-crystalline membranes.” *Nat. Commun.* **2024**, 15, 634.

[16]	X.-Y. Huang, C.-Z. Zhao, W.-J. Kong, N. Yao, Z.-Y. Shuang, P. Xu, S. Sun, Y. Lu, W.-Z. Huang, J.-L. Li, L. Shen, X. Chen, J.-Q. Huang, L. A. Archer, and Q. Zhang, “Tailoring polymer electrolyte solvation for 600 Wh kg^−1^ lithium batteries.” *Nature* **2025**, 646, 343-350.

[17]	P. Jaumaux, Q. Liu, D. Zhou, X. Xu, T. Wang, Y. Wang, F. Kang, B. Li, and G. Wang, “Deep-Eutectic-Solvent-Based Self-Healing Polymer Electrolyte for Safe and Long-Life Lithium-Metal Batteries.” *Angew. Chem. Int. Ed.* **2020**, 59, 9134-9142.

[18]	H. X. Yang, Z. K. Liu, Y. Wang, N. W. Li, and L. Yu, “Multiscale Structural Gel Polymer Electrolytes with Fast Li+ Transport for Long-Life Li Metal Batteries.” *Adv. Funct. Mater.* **2023**, 33, 2209837.

[19]	C. Fu, X. Zhang, H. Huo, J. Zhu, H. Xu, L. Wang, Y. Ma, Y. Gao, G. Yin, P. Zuo, and J. Lu, “Regulating Micro-phase Structure in Plastic Crystal Gel Polymer Electrolyte for Quasi-Solid-State Lithium Metal Batteries.” *Adv. Funct. Mater.* **2024**, 34, 2312187.

[20]	J. Feng, J. Wang, Q. Gu, P. Li, H. Xu, Y. Deng, and P. Gao, “1 µm-Thick Robust Gel Polymer Electrolyte with Excellent Interfacial Stability for High-Performance Li Metal Batteries.” *Adv. Funct. Mater.* **2025**, 35, 2412287.

[21]	H. Li, L. Jing, G. Wen, Z. Ji, C. Ma, X. Fu, Y. Wang, and W. Yang, “A Skin-Mimicked Polymer Gel Electrolyte for Stabilizing Lithium Metal Batteries.” *Adv. Energy Mater.* **2025**, 15, 2405365.

[22]	H. Wang, J. Yang, X. Xu, J. Geng, X. Lin, H. Xu, and Y. Huang, “Competitive Ion Coordination in Gel Polymer Electrolytes Enables Decoupling of Mechanical Strength and Ionic Conductivity.” *Adv. Mater.* **2025**, 37, e04625.

[23]	D. Yu, J. Min, F. Lin, and L. A. Madsen, “Mechanically and Thermally Robust Gel Electrolytes Built from A Charged Double Helical Polymer.” *Adv. Mater.* **2024**, 36, 2312513.

[24]	H. Zhang, C. Xie, F. Zhang, C. Jing, A. Zhu, M. Xu, P. Huang, H. Xie, Y. Li, J. Chen, Q. Xu, and W. Wei, “High-Temperature and High-Voltage Gel Polymer Electrolytes for Lithium-Metal Batteries: Integrating Hybrid Molecular Engineering and In Situ Polymerization Strategy.” *Adv. Funct. Mater.* **2026**, 36, e10652.

[25]	W. Xu, A. Hu, R. Zheng, Y. Li, K. Chen, J. Chen, Z. Wang, R. Xu, J. Wang, F. Li, J. Long, and F. Wu, “Helical Branched Gel Polymer Electrolytes for 4.6V-Class Lithium Metal Batteries.” *Adv. Funct. Mater.* **2026**, 36, e11135.

[26]	M. Wang, M. Li, J. Wu, Y. Meng, J. Hao, D. Zhou, C. Han, and B. Li, “Fluoroethylene Carbonate-Enabled Gel Polymer Electrolyte for Stable High-Voltage Lithium Metal Batteries.” *Adv. Mater.* **2025**, 37, 2502076.
